# Studies on photodegradation process of psychotropic drugs: a review

**DOI:** 10.1007/s11356-016-7727-5

**Published:** 2016-09-30

**Authors:** Jakub Trawiński, Robert Skibiński

**Affiliations:** Department of Medicinal, Medical University of Lublin, Jaczewskiego 4, 20-090 Lublin, Poland

**Keywords:** Photodegradation, Photostability, Photocatalysis, Phototoxicity, Psychotropic drugs, Degradation products

## Abstract

Consumption of psychotropic drugs is still increasing, especially in high-income countries. One of the most crucial consequences of this fact is significant release of them to the environment. Considerable amounts of atypical antipsychotics, benzodiazepines, antidepressants, and their metabolites were detected in river, lake, and sea water, as well as in tissues of aquatic organisms. Their ecotoxicity was proved by numerous studies. It should be noticed that interaction between psychotropic pharmaceuticals and radiation may lead to formation of potentially more toxic intermediates. On the other hand, photo-assisted wastewater treatment methods can be used as an efficient way to eliminate them from the environment. Many methods based on photolysis and photocatalysis were proposed and developed recently; nevertheless, the problem is still unsolved. However, according to recent studies, photocatalysis could be considered as the most promising and far more effective than regular photolysis. An overview on photolytic as well as homogenous and heterogeneous photocatalytic degradation methods with the use of various catalysts is presented. The photostability and phototoxicity of pharmaceuticals were also discussed. Various analytical methods were used for the photodegradation research, and this issue was also compared and summarized. Use of high-resolution multistage mass spectrometry (Q-TOF, ion trap, Orbitrap) was suggested. The combined techniques such as LC–MS, GC–MS, and LC–NMR, which enable qualitative and quantitative analyses in one run, proved to be the most valuable in this case. Assembling of MS/MS spectra libraries of drug molecules and their phototransformation products was identified as the future challenge.

## Introduction

Psychotropic drugs are a wide group of medications used to treat symptoms of mental disorders such as depression, schizophrenia, bipolar disorder, or anxiety disorders (National Institute of Mental Health 2016a).

According to a WHO report (2000), about 10 % of adults at any point in time suffer from mental disorders (Saraceno 2002), but another study showed that lifetime prevalence of such disorders can reach over 25 % (Almeida-Filho et al. 1997). The proportion of disability adjusted life years (DALY) and years of life lived with disability (YLD) caused by neuropsychiatric disorders are 12 and 31 % respectively (Saraceno 2002). Based on the National Survey on Drug Use and Health from 2013, it was estimated that 43.8 million of all adults in the USA had any mental illness in 2012 and 10 million had serious mental illness.In 2013, 15.7 million of all adults in the USA were affected by a major depressive episode over the past 12 months (National Institute of Mental Health 2016d). The WHO Mental Health Action Plan 2013–2020 mentioned that depression accounted for 4.3 % of the global burden of disease, being one of the largest single causes of disability worldwide—11 % of all YLDs (WHO 2013). It should be noticed that in 2000 the first leading cause of YLDs was unipolar depressive disorders.

The second most important mental disease is schizophrenia in which case The Global Burden of Disease 2000 estimates 1.1 % of all DALY and 2.8 % of the total YLD (Saraceno 2002). In 1999, this disorder was found to be the third most disabling condition in comparison to all disabilities associated with physical and mental conditions (Üstün 1999). The National Institute of Mental Health reports 1.1 % 12-month prevalence of schizophrenia in the adult population (National Institute of Mental Health 2016b).

The third large group of mental diseases is anxiety disorders. Lifetime prevalence in the USA is 28.8 % of all adults (National Institute of Mental Health 2016c). General prevalence is estimated on the level of 1.9–5 % of the population in this case. What is more, a high level of comorbidity is observed—about 90 % of patients with generalized anxiety suffer from other mental disorders in their lifetime; the most frequent disorder is major depression (Wittchen 2002).

Taking this into account, antipsychotics, antidepressants, anxiolytics, and the other psychotropic drugs are very widely used nowadays. As proven by the analysis of the Norwegian Institute of Public Health (Drug Consumption in Norway 2010–2014), drugs which belong to the N section of the ATC Classification System (nervous system) are the third best-selling group (420 million defined daily dose, DDD^1^) of drugs in Norway in general. The two most frequently ingested groups amongst them are psycholeptics and psychoanaleptics. Moreover, sales of drugs belonging to these two groups are generally increasing. This statement applies especially to antidepressants—increase from about 12 to 55 DDD/1000 inhabitants/day (from 1990 to 2014) (Norwegian Institute of Public Health 2015). Worth mentioning is the case of antipsychotic drugs, divided into two significantly differing groups: typical and atypical antipsychotics. The first one is represented by pharmaceuticals possessing very high affinity to the dopaminergic receptors which is substantial for treatment of schizophrenia symptoms (but also responsible for adverse effects such as extrapyramidal symptoms or sedation), high affinity to the muscarinic receptors (which causes cholinolytic adverse effects), and relatively low affinity to serotonin receptors. The second one consists of drugs possessing lower affinity to the dopaminergic receptors (however, they are still effective in treatment of positive symptoms of schizophrenia), as well as to the muscarinic receptors, and considerably higher affinity to the serotonin receptors. These properties are responsible for lower probability of appearance of adverse effects after administration of atypical antipsychotics (Kostowski and Herman 2010). Taking into account data from 2011 to 2015 (for Norway), increasing consumption of atypical antipsychotics can be noticed (diazepines, oxazepines, thiazepines, and others such as aripiprazole), while sales of typical antipsychotics are decreasing (this statement refers especially to the phenothiazine derivatives with a piperazine side chain) (Norwegian Institute of Public Health 2016).

Thus, safety of treatment of psychiatric disorders is very crucial. One of the most important factors influencing safety is stability of the prescribed drug. An essential part of stability testing, according to the International Conference on Harmonization of Technical Requirements for Registration of Pharmaceuticals for Human Use (ICH), is photostability testing (ICH 1996). When the European Pharmacopoeia describes over 250 substances as photolabiles (Tonnesen 2004), the importance of such testing becomes clear. Its aim is to prove that a drug substance which is exposed to light does not undergo unacceptable changes (ICH 1996)—resulting not only in loss of activity (degradation) but also in the formation of radicals, energy transfer, or luminescence, which may lead to severe side effects, whose cause could be for example light-induced reaction with endogenous substances (Tonnesen 2004).

Increasing consumption of psychotropic drugs entails their release to the environment through various routes, for instance with patients’ excretions (parent compounds, metabolites, and their conjugates), the pharmaceutical industry, hospital wastewater, medical waste, or agriculture (Evgenidou et al. 2015; Silva et al. 2015). In the case of hospital effluents, the presence of psychiatric pharmaceuticals, as well as their metabolites, is a well-known problem: carbamazepine, its derivatives, and sulpiride were found in the effluents of Takatsuki City hospital in Japan (Azuma et al. 2016), and diazepam, carbamazepine, and their metabolites were detected in effluents from Santa Maria hospital (Brasil) (de Almeida et al. 2015). Similarly, psychotropic drugs were found in effluents from Beijing hospital (China, 22 substances) (Yuan et al. 2013) and from two hospitals in northern Italy (Verlicchi et al. 2012). Psychiatric pharmaceuticals were also detected in river waters, e.g., Yodo River, Japan (Azuma et al. 2015), and the Henares–Jarama–Tajo river system in Spain (Fernández et al. 2010); coastal waters (Moreno-González et al. 2014); and also watershed waters (Esteban et al. 2012), or even drinking water in Serbia (Petrović et al. 2014). Occurrence of psychotropic pharmaceuticals in surface waters is the reason for their presence in tissues of aquatic organisms (Brooks et al. 2005; Ramirez et al. 2007), which cannot be neglected, as toxicity of this group of contaminants to *Ceriodaphnia dubia* (Henry et al. 2004), *Daphnia magna*, *Lymnaea stagnalis* (Lilius et al. 1995), zebra mussels (Fong and Molnar 2008), *Perca fluviatilis* (Brodin et al. 2013), zebrafish (Abreu et al. 2015; Abreu et al. 2015; Abreu et al. 2016; Kalichak et al. 2016; Kalueff et al. 2016), *Oryzias latipes* (Chiffre et al. 2016), and *Gasterosteus aculeatus* (Kellner et al. 2015) was proven. Most of the studies concerning occurrence of pharmaceuticals (including psychotropic substances) focus on wastewater effluents and wastewater treatment plant sludge (Mackuľak et al. 2015b; Mackuľak et al. 2015a; Křesinová et al. 2016). The data concerning occurrence of pharmaceuticals in the environment are assembled in Table 1.

**Table 1:** Occurrence of psychotropic pharmaceuticals in the environmental samples

	Occurrence	Concentration	Reference
Chlorpromazine	WWTP effluent	99 ng L^−1^	(Yuan et al. 2013)
	River water	2.2 ng L^−1^	(Fernández et al. 2010)
Perphenazine	WWTP effluent	3 ng L^−1^	(Yuan et al. 2013)
Fluphenazine	River water	4.1 ng L^−1^	(Fernández et al. 2010)
Sulpiride	WWTP effluent	432 ng L^−1^	(Yuan et al. 2013)
Aripiprazole	WWTP effluent	10.3 ng L^−1^	(Subedi and Kannan 2015)
		1.69 ng L^−1^	
	WWTP sludge	115 ng g^−1^	
		16.8 ng g^−1^	
	WWTP effluent	33 ng L^−1^	(Yuan et al. 2013)
	WWTP sludge	6.68 ng g^−1^	(Subedi et al. 2013)
Clozapine	WWTP effluent	8183 ng L^−1^	(Yuan et al. 2013)
Olanzapine	WWTP effluent	9 ng L^−1^	(Yuan et al. 2013)
	Surface waters	58 ng L^−1a^	(Gracia-Lor et al. 2011)
Quetiapine	WWTP effluent	0.98 ng L^−1^	(Subedi and Kannan 2015)
		4.60 ng L^−1^	
	WWTP sludge	17.8 ng g^−1^	
		21.1 ng g^−1^	
	WWTP effluent	1168 ng L^−1^	(Yuan et al. 2013)
	WWTP sludge	5.41 ng g^−1^	(Subedi et al. 2013)
Risperidone	WWTP effluent	12 ng L^−1^	(Yuan et al. 2013)
	Drinking water	0.34 ng L^−1^	(Snyder 2008)
	WWTP effluent	< 3.1 ng L^−1^	(Mackuľak et al. 2015b)
Ziprasidone	WWTP influent	4 ng L^−1^	(Yuan et al. 2013)
Lorazepam	WWTP effluent	64.2 ng L^−1^	(Subedi and Kannan 2015)
		78.4 ng L^−1^	
	WWTP sludge	0.26 ng g^−1^	
		78.4 ng g^−1^	(Dolar et al. 2012)
	WWTP influent	0.074 μg L^−1^	
	River water	41.27 ng L^−1^	(López-Serna et al. 2010)
	WWTP sludge	11.6 ng g^−1^	(Subedi et al. 2013)
	River water	4 ng L^−1^	(Wu et al. 2015)
	Tap water	562 ng L^−1^	(Esteban et al. 2012)
Bromazepam	WWTP influent	2.3 ng L^−1^	(Wu et al. 2015)
	WWTP effluent	4.2 ng L^−1^	
Alprazolam	WWTP effluent	6.20 ng L^−1^	(Subedi and Kannan 2015)
		4.59 ng L^−1^	
	WWTP sludge	0.61 ng g^−1^	
		0.28 ng g^−1^	
	WWTP effluent	29 ng L^−1^	(Yuan et al. 2013)
	WWTP sludge	10.6 ng g^−1^	(Subedi et al. 2013)
	River water	2.5 ng L^−1^	(Wu et al. 2015)
	Drinking water	2.4 ng L^−1^	
	Tap water	11 ng L^−1^	(Esteban et al. 2012)
Diazepam	WWTP effluent	1.73 ng L^−1^	(Subedi and Kannan 2015)
		2.58 ng L^−1^	
	WWTP sludge	ND	
		0.48 ng g^−1^	
		0.017 μg L^−1^	(Hummel et al. 2006)
	Surface water	0.002 μg L^−1a^	
	River water	6.52 ng L^−1^	(López-Serna et al. 2010)
	WWTP sludge	23 ng g^−1a^	(Peysson and Vulliet 2013)
	WWTP sludge	3.3 ng g^−1^	(Subedi et al. 2013)
	River water	24.3 ng L^−1^	(Wu et al. 2015)
	Drinking water	1.9 ng L^−1^	
Nordiazepam	Surface waters	2.4 ng L^−1^	(Togola and Budzinski 2008)
	WWTP effluent	8.3 ng L^−1^	
	WWTP effluent	17 ng L^−1^	(Esteban et al. 2012)
Oxazepam	WWTP effluent	9.87 ng L^−1^	(Subedi and Kannan 2015)
		7.72 ng L^−1^	
	WWTP sludge	0.86 ng g^−1^	(Dolar et al. 2012)
	WWTP influent	1.60 ng g^−1^	(Hummel et al. 2006)
	WWTP effluent	0.32 μg L^−1^	
	Surface water	0.39 μg L^−1^	
	WWTP effluent	751.1 ng L^−1^	(Yuan et al. 2013)
	WWTP sludge	4.62 ng g^−1^	(Subedi et al. 2013)
	River water	3.3 ng L^−1^	(Wu et al. 2015)
Temazepam	WWTP effluent	0.05 μg L^−1^	(Hummel et al. 2006)
	Surface water	0.023 μg L^−1^	
	River water	1.3 ng L^−1^	(Wu et al. 2015)
	Drinking water	0.2 ng L^−1^	
Tetrazepam	WWTP influent	92 ng L^−1^	(Esteban et al. 2012)
	WWTP effluent	64 ng L^−1^	
Midazolam	WWTP effluent	< 2.9 ng L^−1^	(Mackuľak et al. 2015a, b)
Estazolam	River water	1.2 ng L^−1^	(Wu et al. 2015)
	WWTP influent	6.1 ng L^−1^	
	WWTP effluent	2.9 ng L^−1^	
Meprobamate	WWTP effluent	0.6 ng L^−1^	(Ryu et al. 2010)
	Drinking water	43 ng L^−1^	(Snyder 2008)
Carbamazepine	WWTP effluent	310 ng L^−1^	(Subedi and Kannan 2015)
		268 ng L^−1^	
		83.1 ng g^−1^	
	WWTP sludge	118 ng g^−1^	(Behera et al. 2011)
	WWTP effluent	55 ng L^−1^	(Dolar et al. 2012)
	WWTP influent	0.083 μg L^−1^	(Hummel et al. 2006)
	Surface water	0.025 μg L^−1^	
	WWTP effluent	2956 ng L^−1a^	(Lajeunesse et al. 2012)
	River water	58.43 ng L^−1^	(López-Serna et al. 2010)
	WWTP sludge	50 ng g^−1a^	(Peysson and Vulliet 2013)
	WWTP sludge	23. ng g^−1^	(Subedi et al. 2013)
	River sediment	1.82 ng g^−1^	(Santos et al. 2016)
Amitriptyline	WWTP biosolids	275.4 μg kg^−1^	(Chari and Halden 2012)
	WWTP effluent	128 ng L^−1a^	(Lajeunesse et al. 2012)
	WWTP sludge	273 ng g^−1a^	(Peysson and Vulliet 2013)
	Drinking water	1.4 ng L^−1^	(Togola and Budzinski 2008)
	River water	17 ng L^−1a^	(Kasprzyk-Hordern et al. 2008)
	WWTP influent	3 ng L^−1^	(Wu et al. 2015)
Nortriptyline	WWTP effluent	13 ng L^−1a^	(Lajeunesse et al. 2012)
Clomipramine	WWTP effluent	35 ng L^−1^	(Yuan et al. 2013)
	WWTP effluent	4 ng L^−1^	(Esteban et al. 2012)
	Tap water	27 ng L^−1^	
Venlafaxine	WWTP effluent	480 ng L^−1^	(Subedi and Kannan 2015)
		339 ng L^−1^	
	WWTP sludge	129 ng g^−1^	
		84.2 ng g^−1^	(Gracia-Lor et al. 2012)
	WWTP effluent	0.14 μg L^−1^	
	WWTP effluent	2563 ng L^−1a^	(Lajeunesse et al. 2012)
	WWTP sludge	8.94 ng g^−1^	(Subedi et al. 2013)
	River sediment	26.4 ng g^−1a^	(Santos et al. 2016)
	Tap water	44 ng L^−1^	(Esteban et al. 2012)
Mirtazapine	WWTP effluent	44 ng L^−1a^	(Lajeunesse et al. 2012)
Mianserin	WWTP influent	0.9 ng L^−1^	(Wu et al. 2015)
Doxepin	WWTP effluent	0.17 μg L^−1^	(Hummel et al. 2006)
	Surface water	0.054 μg L^−1^	
	WWTP sludge	60 ng g^−1a^	(Peysson and Vulliet 2013)
	River water	0.8 ng L^−1^	(Wu et al. 2015)
Bupropion	WWTP effluent	67.4 ng L^−1^	(Subedi and Kannan 2015)
		34.1 ng L^−1^	
	WWTP sludge	23.7 ng g^−1^	
		12.5 ng g^−1^	
	WWTP effluent	0.191 μg L^−1a^	(Metcalfe et al. 2010)
	WWTP sludge	1.07 ng g^−1^	(Subedi et al. 2013)
Paroxetine	WWTP biosolids	61. μg kg^−1^	(Chari and Halden 2012)
	WWTP effluent	12 ng L^−1a^	(Lajeunesse et al. 2012)
	WWTP effluent	0.016 μg L^−1a^	(Metcalfe et al. 2010)
	WWTP sludge	89 ng g^−1a^	(Peysson and Vulliet 2013)
	River sediment	3.1 ng g^−1a^	(Santos et al. 2016)
Sertraline	WWTP effluent	62.8 ng L^−1^	(Subedi and Kannan 2015)
		24.5 ng L^−1^	
	WWTP sludge	1490 ng g^−1^	
		862 ng g^−1^	(Chari and Halden 2012)
	WWTP biosolids	458 μg kg^−1^	
	WWTP effluent	34 ng L^−1a^	(Lajeunesse et al. 2012)
	WWTP effluent	0.034 μg L^−1a^	(Metcalfe et al. 2010)
	WWTP sludge	3834 ng g^−1a^	(Peysson and Vulliet 2013)
	WWTP sludge	56.7 ng g^−1^	(Subedi et al. 2013)
	River sediment	7.89 ng g^−1a^	(Santos et al. 2016)
Citalopram	WWTP effluent	280 ng L^−1^	(Subedi and Kannan 2015)
		150 ng L^−1^	
	Sludge	283 ng g^−1^	
		170 ng g^−1^	
	WWTP effluent	223 ng L^−1a^	(Lajeunesse et al. 2012)
	WWTP effluent	0.223 μg L^−1a^	(Metcalfe et al. 2010)
	WWTP sludge	26.8 ng g^−1^	(Subedi et al. 2013)
	River sediment	14.4 ng g^−1a^	(Santos et al. 2016)
Escitalopram	WWTP sludge	313 ng g^−1a^	(Peysson and Vulliet 2013)
Fluoxetine	WWTP effluent	20 ng L^−1a^	(Lajeunesse et al. 2012)
	Drinking water	2.74 ng L^−1^	(López-Serna et al. 2010)
	WWTP effluent	0.091 μg L^−1a^	(Metcalfe et al. 2010)
	WWTP sludge	212 ng g^−1a^	(Peysson and Vulliet 2013)
	River sediment	7.78 ng g^−1a^	(Santos et al. 2016)
	River water	0.4 ng L^−1^	(Wu et al. 2015)
Fluvoxamine	WWTP effluent	3.9 ng L^−1a^	(Lajeunesse et al. 2012)
Trazodone	River sediment	5.6 ng g^−1^	(Santos et al. 2016)

^a^Highest measured concentration (in other cases mean concentrations are shown)

From the environmental point of view, the interaction of psychotropic pharmaceuticals with visible and UV radiation has both positive and negative sides. On the one hand, photodegradation can be used as an effective method of wastewater treatment. Plenty of studies discussing direct (Boreen et al. 2003; Lin and Reinhard 2005; Wawryniuk et al. 2015) and indirect (Sousa et al. 2013b; Yu et al. 2015) (homogeneous and heterogeneous photocatalysis with the use of a very wide variety of catalysts) photolysis are available. However such methods are effective in laboratory scale, they often need to be adjusted to the larger, industrial scale. Moreover, methods based on the advanced oxidation processes are still not routinely used in wastewater treatment plants. Another potentially positive aspect of the photolysis process is decomposition of psychotropic drugs released to the environment and, as a consequence, decrease of their toxicity (this statement applies only to the photolabile pharmaceuticals). On the other hand, this process may lead to the formation of intermediates which can be more toxic than the parent compound (Isidori et al. 2005).

In our opinion, the main future challenge we must take up in order to have control over psychotropic pharmaceuticals (as well as the other groups of drugs) is introduction of the investigation of the radiation–molecule interaction during the design stage of the new drugs. A very important and often neglected question is exhaustive photostability testing of pharmaceuticals present on the market. Such studies should absolutely include structural elucidation and, preferably, toxicity assessment of the photoproducts. Another substantial issue is to start to treat advanced oxidation processes as the routinely applied stage in wastewater treatment.

## Photostability testing of drugs

The ICH Q1B Guideline describes the photostability testing of new drug substances and products. Confirmatory studies provide information to be applied to handling, packaging, labeling, and forced degradation testing which allow assessing the photosensitivity of a substance and its photodegradation pathway. The guideline does not define certain exposure conditions (ICH 1996).

Two recommended light sources are described in Q1B – D65 (simulating outdoor daylight) or ID65 (simulating indoor indirect window glass-filtered daylight) as option 1 and simultaneous exposition on a cool white fluorescence lamp (producing an output similar to that specified by ISO 10977) and near UV fluorescent lamp (spectral distribution from 320 to 400 nm with a maximum energy emission between 350 and 370 nm) as option 2 (ICH 1996). Because option 2 simulates indoor lighting, it is not equivalent to option 1 (Baertschi et al. 2013). Overall illumination of the sample is also described in Q1B—it should be not less 1.2 million lx h; integrated energy of near ultraviolet should be not less than 200 W h m^−2^. Dark controls and a validated chemical actinometric system or calibrated radiometer should also be applied. As an actinometer for monitoring exposure to the near UV fluorescent lamp, ICH suggests the use of 2 % *w*/*v* aqueous solution of quinine monohydrochloride dihydrate. The radiation dose is calculated from change in quinine solution absorbance at 400 nm (ICH 1996). Some tests proved that quinine actinometry is concentration dependent, and the calibration curve may vary depending on the location of the solution in the photostability chamber, so ICH guidelines should not be generalized (de Azevedo Filho et al. 2011).

Data obtained in photostability testing should be used to apply proper handling, packaging, and labeling of a drug substance and predict its possible side effects (connected with light exposure) and drug delivery system (e.g., it should be applied to infusion solutions which are often stored in transparent bottles—the consequence is long exposure of drug substance on indoor light) (Tonnesen 2004; Baertschi et al. 2013).

Although the ICH Q1B Guideline is implemented in Europe, USA, and Japan (Tonnesen 2004), it may not cover all steps in the drug supply chain, for example, dilution and reconstitution before administration. Moreover, the proved photostability of the substance itself is not evidence that the formulation after addition of excipients will be stable. Photodegradation can be also induced by impurities (e.g., iron compounds in citrate buffers) (Baertschi et al. 2013).

Some authors suggest the application of different guidelines, however sometimes based on those recommended by ICH. For example, Singh and Bakshi proposed the introduction of a second exposure time. Whether the acceptable change cannot be observed after exposure of 1.2·10^6^ lx h, it should be increased to 6.0·10^6^ lx h (Singh and Bakshi 2000).

Amongst the psychotropic drugs, the most studied group, from the photostability testing point of view, is antipsychotics. Photolability of phenothiazine derivatives is a well-established fact—irradiation of chlorpromazine with UV-C (254 nm) and UV-A (365 nm) caused its significant loss (Prohotsky et al. 2014); however, according to another study, after application of ICH-recommended overall illumination, only 4.5 % of the drug was degraded. In the case of trifluoperazine (the same study), degradation of 9.5 % of the substance was observed (Shetti and Venkatachalam 2010). Irradiation of trifluoperazine with sunlight and UV light yielded the formation of two phototransformation products (Subbareddy and Divakar 2016). Stability studies of levomepromazine in mixtures with midazolam and morphine revealed that this antipsychotic agent is susceptible to white fluorescent light (complete decomposition or about 3 % of the initial concentration left, depending on the examined mixture; other drugs were more stable) (Fernandez-Campos et al. 2013). Fluorescent light was also applied for stability testing of dixyrazine, which turned out to be stable at these conditions. Degradation was observed after application of the CPS Suntest stability chamber (Kopelent-Frank and Mittlböck 1996). Two photoproducts were detected after irradiation of polyethylene glycol solution of perphenazine with fluorescent light (Li et al. 2005), and over 50 in the case of prochlorperazine irradiated with 365 nm radiation (Lew et al. 2011). Distinct photolysis was also observed in the case of fluphenazine (41 % loss after 5 h of 254 nm irradiation) (Belal et al. 2008). Amongst butyrophenone derivatives, photostability studies were conducted only on haloperidol, which underwent complete decomposition after 21 days exposure to daylight (transparent glass bottle) (Driouich et al. 2001). Another study reported the formation of one product after irradiation with UV light and sunlight (Mennickent et al. 2008). No degradation was observed in the case of indole derivative, sertindole, under UV irradiation (El-Ragehy et al. 2014). Many papers discussing photostability of ziprasidone were published. According to two of them, ziprasidone hydrochloride is photostable under UV–VIS irradiance (Singh et al. 2007; Zakowiecki and Cal 2012); however, the second one reported photolability of ziprasidone free base. Dependence of susceptibility to UV-C irradiation on the applied solvent was reported by Walash et al.—31 % of ziprasidone was degraded after 24 h irradiation of aqueous solution, while only 9 % in the case of methanolic solution (Walash et al. 2011a). The number of photoproducts was also dependent on applied wavelength—12 in case of use of UV-C and only one in case of UV-A (Skibiński 2012a). Chlorprothixene is the only representative of the thioxanthene group that was submitted for photostability testing. In this case, the Suntest photostability chamber was used, and after 6 h of irradiation more than 50 % of the drug was decomposed (Kopelent-Frank and Mittlböck 1996). Pimozide turned out to be not susceptible to sunlight (5 h of irradiation) (Manjula and Ravi 2013). Most of the studied atypical antipsychotics were photostable. Clozapine (Perks et al. 2012), risperidone (Svirskis et al. 2011), and paliperidone (Bindu et al. 2012) were stable under the ICH conditions. Paliperidone did not undergo photolysis under UV radiation (Sherje and Londhe 2015). Quetiapine is also photostable under ICH conditions, as well as UV-C radiation (Narendra et al. 2011; Kumar et al. 2013), asenapine under UV and sunlight (Chhalotiya et al. 2012; Patel et al. 2015), levosulpiride under UV radiation (Pal et al. 2016), and aripiprazole under ICH conditions and UV-C irradiation (Srinivas et al. 2008; Narayana and Chandrasekhar 2012; Pai and Dubhashi 2013). Exposure of amisulpride to sunlight for 48 h resulted in 9.5 % degradation (Dabhi et al. 2013). According to Talluri et al. (2014), the solid state of zotepine was stable under white fluorescent light (after 72 h), while irradiation of solution caused 25 % degradation within 3 h. Zotepine in its solid state was more susceptible to sunlight (2.3 % degradation after 8 h). No degradation was observed in the case of olanzapine under UV-C irradiation (Shah et al. 2008; Krishnaiah et al. 2011), but according to another study (Pathak and Rajput 2009), this substance undergoes phototransformation in neutral, acidic, and basic solutions with creation of five photoproducts.

Amongst antidepressants, photostability of duloxetine is probably most studied; however, the reports are contradictory. According to some research statements, this substance is stable (or practically stable) under ICH conditions and sunlight (Chhalotiya et al. 2010; Raman et al. 2010; Rao et al. 2010; Kumar et al. 2012; Veera et al. 2012), and even UV-C irradiation (95 % of substance left after 48 h) (Srinivasulu et al. 2009). According to other studies, duloxetine undergoes photochemical transformation under UV-C radiation (three products) (Gomes et al. 2010) and sunlight (Patel et al. 2009), which caused complete degradation of the substance (Patel et al. 2010). Antidepressant agents that can be considered as photostable are desipramine (ICH conditions) (Thiyagarajan et al. 2008), citalopram (ICH conditions and UV radiation) (Sharma et al. 2011; Tapkir et al. 2016), escitalopram (wavelength 265 nm) (Kakde et al. 2013), sertraline (UV-C) (Walash et al. 2011b), agomelatine (also UV-C) (El-Shaheny 2014), moclobemide (Patel et al. 2008), and paroxetine (Venkatachalam and Chatterjee 2007) (sunlight in both cases). Bupropion (Bansal et al. 2013) and dosulepin (Reddy et al. 2014) are unstable under ICH conditions. Photostability of maprotiline was investigated under doubled ICH conditions (400 W m^−2^ and 2.4 * 10^6^ lx h, the drug was unstable) (Đurić et al. 2015). Venlafaxine under UV radiation was almost stable (3.95 % of the substance decomposed after 10 h of irradiation) (Rao et al. 2015). Exposure to UV-C radiation causes degradation of tianeptine (four photoproducts) (Khedr 2007) and mianserin (Sfair et al. 2012b). Fluvoxamine was unstable under visible and UV radiation, especially in solution (Souri et al. 2015). Fluoxetine irradiated in the photostability chamber for 15 days was susceptible to radiation, especially in basic solution (two photoproducts were detected) (Pathak and Rajput 2009).

Photostability testing on benzodiazepines revealed that this group of drugs is rather susceptible to photodegradation. Both chlordiazepoxide (Pathak et al. 2010) and alprazolam (Cabrera et al. 2005) decomposed under ICH conditions; however, the first one was more photolabile in basic solution, while the second one in acidic. Clobazam in solid state was rather stable (especially under visible radiation), but in the case of its solution, UV radiation caused significant decomposition (Souri et al. 2014). Complete degradation with formation of *N*-desmethyldiazepam was reported after exposure of clorazepate dipotassium salt to sunlight for 4 days (Burana-Osot et al. 2015). Amongst non-benzodiazepine anxiolytics, hydroxyzine (Sher et al. 2015) and meprobamate (Karthikeyan et al. 2010) are practically photostable (sunlight and UV–VIS radiation exposure respectively). Irradiation of etifoxine with a mercury lamp for 24 h resulted in degradation of 20 % of substance and formation of two photoproducts (Djabrouhou and Guermouche 2014). One photoproduct was detected in the case of buspirone after 30 days of daylight irradiation (Khedr and Sakr 1999). Investigation of photostability of carbamazepine^2^ (methanolic solution, 100 μg mL^−1^) showed that this compound is moderately susceptible to solar irradiation (natural sunlight). After 4 h of experiment, 9.25 % of the substance was decomposed and one phototransformation product was detected (Rajadhyaksha et al. 2007). On the contrary, oxcarbazepine irradiated (100 μg mL^−1^, solvent consisting of acetonitrile and methanol) with UV-C (254 nm) for 10 days was completely stable (Pathare et al. 2007).

As it can be seen, requirements concerning photostability testing are based on a 20-year-old guideline which clearly needs to be revised. Firstly, an improved guideline should cover the whole drug supply chain. The next issues are to specify and clarify the description of radiation sources used for drug testing and to correct requirements involving exposure and overall illumination of the sample (solutions of these problems were suggested by Baertschi et al. (2010)). An essential question is also introduction of photostability testing of active pharmaceutical ingredients in the presence of formulation ingredients.

## Photodegradation of pharmaceuticals

Photodegradation, the process of decomposition induced by light (near ultraviolet and visible radiation), depends on the absorption spectrum of the drug molecule. Generally, whether the substance does not absorb radiation over 290 nm, direct photodegradation under solar radiation cannot occur, because of absorptive properties of the ozone layer (Tonnesen 2004). Of course, if the source of artificial light (e.g., low-pressure mercury lamp) is applied, photodegradation of such compound may be possible.

**Fig. 1:**
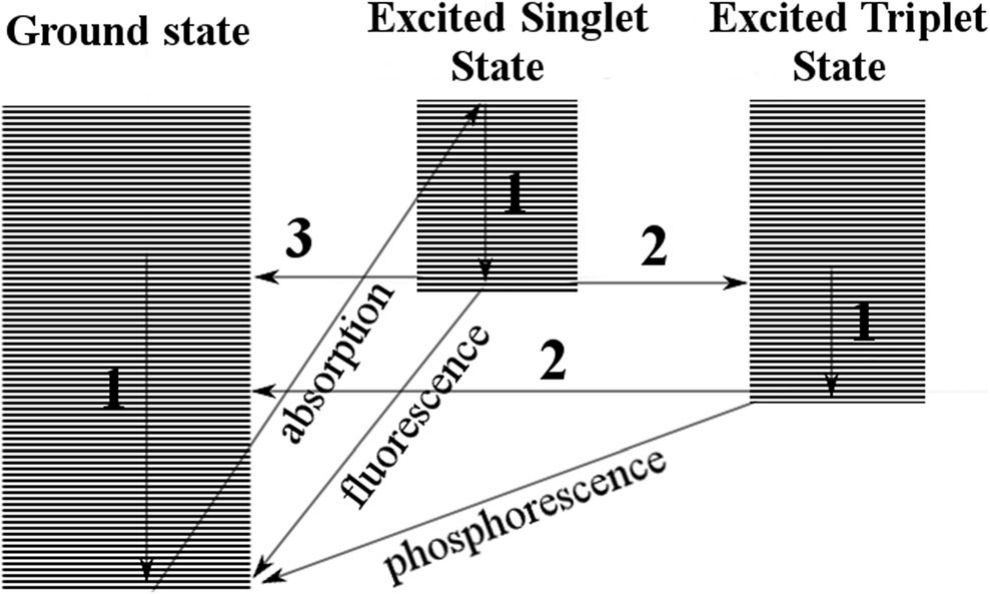
Transitions after absorption of energy by molecule (*1* vibrational relaxation, *2* intersystem crossing, *3* internal conversion); based on Tonnesen (2004)

After absorption of energy by a molecule, an electron is excited from the ground state to the excited state, which is less stable and, therefore, usually rapidly deactivated. The process of deactivation leads to dissipation of energy via various transitions such as internal conversion (return to ground state), fluorescence, photoionization (removal of an electron from a molecule), phosphorescence, or intersystem crossing (transition to a metastable excited triplet state) (Fig. 1) (Tonnesen 2004).

There are numerous factors influencing the photodegradation process: radiation source, pH of substance solution, applied solvents, presence of organic (for instance humic acids) and inorganic matrix or quantum yield (φ) of substance photochemical reaction. This last parameter is characteristic for each compound and is independent of other listed factors (i.e., it should be dependent only on properties of the studied molecule; however, reported values of quantum yield for the same compound often vary, which is problematic; moreover, in practice, it is also dependent on pH) (Challis et al. 2014). It is defined as the ratio of the number of molecules reacted to the number of photons absorbed (Eq. 1).

1 $$ \varphi =\frac{\mathrm{number}\kern0.5em \mathrm{of}\kern0.5em \mathrm{molecules}\kern0.5em \mathrm{reacted}/\mathrm{unit}\kern0.5em \mathrm{volume}/\mathrm{unit}\kern0.5em \mathrm{time}}{\mathrm{number}\kern0.5em \mathrm{of}\kern0.5em \mathrm{photons}\kern0.5em \mathrm{absorbed}/\mathrm{unit}\kern0.5em \mathrm{volume}/\mathrm{unit}\kern0.5em \mathrm{time}}\kern.1em \left[ mol\kern.3em  einstei{n}^{-1}\right] $$

Thus, if we know that quantum yield is constant, the rate of direct photolytic reaction depends only on the number of absorbed photons (Tonnesen 2004). Quantum yield is usually measured with the use of chemical actinometers and calculated from Eq. (2) for the polychromatic radiation source or Eq. (3) for the monochromatic radiation source (*φ* quantum yields of target chemical (*c*) and actinometer (*a*), *k*_p_ direct photodegradation rate constant of chemical (*c*) and actinometer (*a*), *ε*_*λ*_ molar absorption coefficient of chemical (*c*) and actinometer (*a*), *I*_*λ*_ light intensity).

2 $$ {\varphi}_c=\left(\frac{k_{\mathrm{p}c}}{k_{\mathrm{p}a}}\right)\left(\frac{\varSigma {I}_{\lambda }{\varepsilon}_{a\lambda }}{\varSigma {I}_{\lambda }{\varepsilon}_{c\lambda }}\right){\varphi}_c $$

3 $$ {\varphi}_c=\left(\frac{k_{\mathrm{p}c}}{k_{\mathrm{p}a}}\right)\left(\frac{\varepsilon_{a\lambda }}{\varepsilon_{c\lambda }}\right){\varphi}_a $$

As was pointed out, pH influences the value of quantum yield. It is the consequence of fact, that when solved, molecules always exist in equilibrium with their protonated form (and each of these species have different quantum yields). The apparent quantum yield for such chemical system, especially measured at a pH close to compound p*K*_a_, is unstable and may be unreliable (Challis et al. 2014). A study by Ge et al. shows dependence of degradation rates on quantum yields of fluoroquinolones. Ciprofloxacin, a compound with the lowest value of quantum yield (7.98 · 10^−2^), is characterized by the longest half-life time (1.24 min), while enrofloxacin (the highest value of quantum yield, 1.54 · 10^−1^) possesses the shortest half-life time (0.56 min) (Ge et al. 2015). The influence of solution pH on the photodegradation rate of other fluoroquinolones sarafloxacin and difloxacin (isoelectric points at pH 7.31 and 6.15 respectively) was explored by Prabhakaran et al. In the case of difloxacin, the highest degradation rate was observed at neutral pH (close to its isoelectric point), but sarafloxacin was least stable at pH = 9 (Prabhakaran et al. 2009). Ciprofloxacin was most susceptible to photolysis at slightly basic pH (close to its isoelectric point) (Torniainen et al. 1996). Khaleel et al. investigated the influence of various parameters on photodegradation of desipramine. The highest degradation rate was observed at pH = 9, when desipramine is in its unprotonated form. This fact was attributed to higher photolability of free base than protonated forms (Khaleel et al. 2016). The photodegradation rate of synthetic estrogen, ethinylestradiol (p*K*_a_ = 10.5), was highest at pH = 11 (unprotonated molecules, highest susceptibility to radiation) (Ren et al. 2016).

Presence of inorganic, or, especially, organic matrix usually changes the photodegradation rate, as a consequence of indirect photolysis. This process concerns also compounds not absorbing photons of applied wavelengths. However, most of compounds undergo direct and indirect photolysis at the same time. In such case, the term “indirect photolysis constant” rather refers to the summed (direct and indirect) constants. Species responsible for indirect photolysis include inorganic ions such as nitrates, carbonates, iron, and dissolved organic matter (DOM) (Challis et al. 2014) whose role is most studied. The mechanism of DOM “activation” is in general similar to molecule excitation in direct photolysis process. The singlet-excited state of DOM may return to the ground state or transit via intersystem crossing to the excited triplet state. Then, energy transfer between excited DOM and the molecule of interest or oxygen takes place, and DOM returns to its ground state. The excited oxygen molecule decomposes into reactive oxygen species (ROS): excited state of bimolecular oxygen and superoxide anion radical. The second one decomposes into H_2_O_2_ and then into hydroxyl radicals. All of ROS react with drug molecules, causing their degradation. Decomposition may also occur after energy transfer from excited DOM (Yan and Song 2014). The presence of DOM generally enhances the degradation rate; however, in some cases, the opposite situation is observed (e.g., ketoprofen) (Lin and Reinhard 2005). In a study on ciprofloxacin, addition of various DOM (humic and fulvic substances) was investigated. Their influence on the degradation rate was similar (ninefold increase compared to direct photolysis) (Porras et al. 2016). The presence of DOM also significantly increases photodegradation rates of tramadol, venlafaxine, their demethylated metabolites, and lidocaine—5, 8, 15, 13, and 59 times, respectively, in comparison with direct photolysis (Rúa-Gómez and Püttmann 2013). Interesting findings were reported in a study on mianserin by Wawryniuk et al. This compound undergoes direct photolysis under UV–VIS radiation and is resistant to VIS radiation alone. After addition of synthetic humic water, application of VIS radiation was sufficient to decompose mianserin (the degradation rate was higher than in the direct photolysis UV–VIS experiment) (Wawryniuk et al. 2015). Humic substances also increased the photodegradation rate (four times) of ethinylestradiol (Ren et al. 2016).

Photolysis reactions usually fit the first-order kinetics model (*c*_*t*_ concentration of the chemical after time *t*, *c*_0_ initial concentration of the chemical, *k*_1_ first-order kinetic constant, *k*_0_ zero-order kinetic constant, *t* time, Eq. 4). However, this statement can be true only with reference to a low concentration of the studied compound (i.e., low value of solution absorbance). In such case, the photoreaction rate is directly proportional to the concentration of the substance. In the opposite case, the photochemical reaction rate is limited by the radiation intensity, not by compound concentration. If solution absorbance is too high, the order of photodegradation changes from first to zero (the concentration of the substance decreases linearly with time, Eq. 5) (Tonnesen 2004).

3 $$ \ln \left({c}_t\right)= \ln \left({c}_0\right)-{k}_1t $$

4 $$ {c}_t={c}_0-{k}_0t $$

In most of the photodegradation experiments concerning pharmaceuticals, the first-order kinetics model was observed, for instance in the case of difloxacin and sparfloxacin (Prabhakaran et al. 2009), nitrendipine (Tipre and Vavia 2001), labetalol (Andrisano et al. 2001a), or duloxetine (Gomes et al. 2010). Findings concerning photodegradation of mianserin are contradictory. According to Wawryniuk et al., photolysis of mianserin in aqueous solution was described by first-order kinetics (UV–VIS radiation) (Wawryniuk et al. 2015), while Sfair et al. reported zero-order kinetics (UV-C radiation, methanolic solution) (Sfair et al. 2012b). The difference between findings of these studies can be attributed to the different initial concentration of mianserin (20 and 80 mg L^−1^ respectively).

The photoreactivity of several chromophores is well established. In the case of halogenated aromatic and heteroaromatic compounds, the common reaction is photodehalogenation, for example, defluorination of fluoroquinolones (Soldevila and Bosca 2012; Ge et al. 2015; Porras et al. 2016). Aryl cations generated during this process possess a high affinity to biomolecules (especially to amino acids tryptophan and tyrosine). Consequences of this process are photoallergic properties of fluoroquinolones (Soldevila et al. 2014). Another pharmaceutical undergoing photodehalogenation is antifungal itraconazole which loses its chlorine atoms under UV-B radiation. The loss of chlorine may be followed by intramolecular rearrangement or reaction with biomolecules (Nardi et al. 2013). An anti-inflammatory drug diclofenac also undergoes photodehalogenation, followed by formation of chlorocarbazole, which is probably responsible for phototoxic properties of this pharmaceutical (Encinas et al. 1998a). Some of drugs, e.g., tiaprofenic acid (aliphatic carboxyl group) (Encinas et al. 1998b) or rufloxacin (Condorelli et al. 1999), are susceptible to photo-induced decarboxylation. Aniline derivatives, such as aminosalicylic acid, can undergo photo-oxidation (Jensen et al. 1992). Compounds containing phenol groups are very susceptible to photo-oxidation. Irradiation of 17-*β*-estratiol and estrone results in benzene ring oxidation, cleavage, and formation of carbonyl compounds (Liu and Liu 2004). In the case of estradiol, addition of Rose Bengal as a sensitizer results in formation of catechol and cyclohexadienone derivatives (then further oxidation takes place) (Díaz et al. 2009). The enol group of doxycyclin is oxidized to a carbonyl group, as an effect of direct photolysis (Yuan et al. 2011). Oxidation is also a common reaction amongst sulfur-containing pharmaceuticals like phenothiazine derivatives (formation of sulfoxide, but also nitroxide, e.g., in the case of chlorpromazine) (Kochevar 1981). Irradiation of diltiazem with UV-A and UV-B radiation similarly results in formation of sulfoxide (Andrisano et al. 2001b). Photo-induced addition of oxygen takes place in the case of naphtoquinone derivative, menadion (vitamin K), with formation of an epoxide, as a result (Vire et al. 1980). In the case of tetrahydropyridine derivatives (e.g., nifedipine, nitrendipine), exposure to radiation causes dehydrogenation to pyridine derivative (Sadana and Ghogare 1991; Tipre and Vavia 2001). Nitro derivatives of aromatic and heteroaromatic compounds can undergo photoreduction reactions. For example, hypnotic drug flunitrazepam irradiated with 300 nm radiation transforms into its amino derivative (Givens et al. 1986), and in the case of antiandrogen drugs, the flutamide nitro group is reduced to the nitroso group (in the presence of cyclodextrins) or substituted with the phenol group (in water) (Sortino et al. 2001). Another group of photo-induced reactions is composed of isomerization and rearrangement. Heteroaryl-conjugated alkenes can be isomerized between *E* and *Z* forms. Examples of such compounds are thioxanthene antipsychotics, for instance chlorpothixene (Kopelent-Frank and Mittlböck 1996), or tricyclic antidepressant, doxepin (Tammilehto et al. 1982). Photoisomerization also takes place in the case of the alkoxyimino group of monobactam antibiotic, aztreonam (*syn-anti*-isomerization) (Fabre et al. 1992). Photorearrangement is observed after irradiation of the anti-inflammatory agent, benzydamine (detachment of the aliphatic chain from the oxygen atom followed by attachment to the nitrogen atom, and formation of cyclic ketone) (Vargas et al. 1993). Heteroaromatic rings such as imidazoline (e.g., naphazoline; Sortino et al. 1999), diazine (e.g., barbiturates; Jochym et al. 1988), azetidine (beta-lactam antibiotics and derivatives; Albini et al. 1995), or diazepine (e.g., diazepam) also undergo rearrangement or cleavage (Tonnesen 2004). Because of stabilization of the benzylic radical and cation, aromatic compounds can undergo benzylic position fragmentation. An example of such reaction is cleavage of 2-propionic acids (e.g., anti-inflammatory drug, naproxen) (Moore and Chappuis 1988). Photo-induced fragmentation occurs also in the case of phenethylamines (in the presence of α-hydroxy group, e.g., β-blocker, labetalol; Andrisano et al. 2001a) or heterocycles containing β-amino group (e.g., quinine; Epling and Yoon 1977). Amongst ketones, α-cleavage is a common photoreaction. An example of pharmaceuticals undergoing such transformation are glucocorticosteroids (e.g., cortisone and hydrocortisone) (Tonnesen 2004) and anthracycline derivative antineoplastic daunorubicin (Li and Chignell 1987). Carbonyl compounds undergo also hydrogen abstraction (Tonnesen 2004). Aliphatic amines can also undergo carbon–nitrogen bond cleavage (dealkylation reaction), for instance photodealkylation of chloroquine (Nord et al. 1991).

As it can be seen, photoreactiviy of pharmaceuticals is rather common; therefore, irradiation can lead to the formation of compounds whose structure, properties, or potential toxicity are unknown and should be assessed. On the other hand, photo-induced degradation can be used in wastewater treatment.

## Environmental aspects of photodegradation

Nowadays, pharmaceuticals are still considered as emerging pollutants, because of lack of regulations or directives (Rivera-Utrilla et al. 2013). There are various routes by which pharmaceuticals can reach the environment: excretions, wastewater effluents, medical waste, industrial waste from manufacturing, or agriculture (Silva et al. 2015). Processes that drugs undergo after administration lead to the formation of metabolites which are excreted and thus should be also considered as pollutants. About 30–90 % of administered drugs are excreted as active substances which may undergo chemical, biological, or physical transformation (Rivera-Utrilla et al. 2013). The presence of pharmaceuticals and their metabolites in rivers, lakes, seas, and, of course, wastewater and municipal solid waste (MSW) was proved by numerous studies (Loos et al. 2007; Musson and Townsend 2009; Fernández et al. 2010; Munaron et al. 2012; Moreno-González et al. 2014; Subedi and Kannan 2015). For example, the concentration of antipsychotic and antihypertensive drugs in Albany area in New York varies between 0.98 ng L^−1^ (for quetiapine) and 1220 ng L^−1^ (for atenolol) in wastewater, and between 0.26 ng L^−1^ (for lorazepam) and 1490 ng L^−1^ (for sertraline) in sludge (Subedi and Kannan 2015). The total access of pharmaceuticals into Mar Menor lagoon in Spain was assessed 11.3 kg annually. According to the same study, pharmaceuticals detected at the highest concentrations (over 1000 ng L^−1^) were azithromycine, clarithromycine, valsartan, paracetamol, and ibuprofen, while the most persistent were lorazepam, carbamazepine, diclofenac, phenazone, torasemide, or trimethoprim (Moreno-González et al. 2014).

Psychotropic pharmaceuticals were found in tissues of aquatic organisms—fluoxetine, sertraline, and their metabolites accumulated mainly in brain tissue of fish (Brooks et al. 2005) and carbamazepine and norfluoxetine occurrence was reported in muscle tissues of *Lepomis* sp. (Ramirez et al. 2007). Exposure to pharmaceuticals and their biologically active metabolites may affect aquatic organisms (Oaks et al. 2004; Kidd et al. 2007). Psychotropic drugs with affinity to the nervous and endocrine systems belong to the group of the most ecotoxic pharmaceuticals (Calisto and Esteves 2009). For example, SSRIs in general can influence serotonin levels in invertebrates and fish; fluoxetine affects reproduction; italopram increases mortality in *C. dubia* (Henry et al. 2004); paroxetine, fluvoxamine, and norfluoxetine induce spawning in male zebra mussels (Fong 1998; Fong and Molnar 2008); and venlafaxine changes the immunocompetence of *L. stagnalis* (Lilius et al. 1995). Assessment of benzodiazepine ecotoxicity revealed that diazepam acts as a growth inhibitor of *D. magna* (Lilius et al. 1995), and oxazepam affects the behavior and feeding rate of *P. fluviatiis* (Brodin et al. 2013). Pharmaceuticals and their metabolites can also show phytotoxic properties, e.g., β-blockers and their metabolites inhibit the photosynthesis rate in green algae (Escher et al. 2006).

Conventional wastewater treatment consists of two steps: physico-chemical (primary treatment) and biological (secondary treatment) (Rivera-Utrilla et al. 2013), and it is often not effective enough in pharmaceutical removal (Glassmeyer and Shoemaker 2005; Fatta-Kassinos et al. 2011a; Vieno and Sillanpää 2014; Silva et al. 2015). One of the newest groups of wastewater treatment methods is advanced oxidation processes (AOPs) based on formation of active oxygen species (AOS), mainly radicals such O_2_^−·^, HO^·^_2_, and HO^·^ which is the strongest oxidizer (Rivera-Utrilla et al. 2013; Silva et al. 2015). AOPs can be divided into two main groups: homogeneous and heterogeneous (depending on the number of applied phases). Each of them can be performed with or without radiation (Silva et al. 2015).

The efficiency of direct photodegradation depends on the absorption spectrum and process quantum performance (Silva et al. 2015). As it was mentioned above, solar irradiation itself can be applied only in the case of pharmaceuticals absorbing radiation at wavelengths over 290 nm. Direct photodegradation of other compounds can be performed using shorter wavelengths, e.g., 254 nm (UV-C radiation), although application of such conditions is criticized as irrelevant with reference to environmental studies (Challis et al. 2014). Nevertheless, UV-C radiation (used in germicidal lamps and disinfection systems) can be used during wastewater treatment. Its usefulness for decomposition of pharmaceuticals as well as other emerging contaminants was studied and reported by several papers (for instance photodegradation of flupentixol Maquille et al. 2010, olanzapine Karpińska et al. 2012, fluoxetine, fluvoxamine, and other fluorinated compounds Hidaka et al. 2013). High efficiency of UV-C application for large groups of pharmaceuticals was also reported by Kim and Tanaka (2009) and Pereira et al. (2007). According to several studies, irradiation alone may be used as an effective degradation method for some compounds (Boreen et al. 2003; Lin and Reinhard 2005; Wawryniuk et al. 2015), unlike others that mention that some compounds do not undergo direct photodegradation, e.g., venlafaxine (Santoke et al. 2012). Comparison of different types of mercury lamps demonstrates that use of a polychromatic medium-pressure UV lamp results in a higher degradation rate than a monochromatic low-pressure UV lamp (Tixier et al. 2003). Even if a molecule is affected by irradiation, and thus undergoes the process of direct photodegradation, it does not mean that the contamination problem is solved—products of photochemical reactions can be more toxic than parent compounds, e.g., irradiation of naproxen generates possibly genotoxic derivatives (Isidori et al. 2005) and photolytic degradation of triclosan leads to formation of dioxin (DCDD) which is supposed to be more stable than the parent compound (Aranami and Readman 2007).

More satisfactory effectiveness of photodegradation could be usually achieved using indirect photodegradation methods, based on irradiation-dependent formation of reactive oxygen species, mainly the hydroxyl radical—the second most powerful oxidizer, after fluorine (2.8 V versus normal H electrode) (Santoke et al. 2012; Klamerth et al. 2012). The combination of UV radiation and hydrogen peroxide gives the hydroxyl radical as a product which substantially increased the degradation rate of nortriptyline in comparison to UV alone (Benitez et al. 2013). A large study on degradation of 40 pharmaceuticals proved that the UV/H_2_O_2_ method effectively removes most of the drugs, but some of them (cyclophosphamide, metformin) can resist such treatment (Wols et al. 2013). Organic compounds—dissolved organic matter (DOM) present in wastewater and surface waters—can act as AOS generators. Studies on photodegradation of mianserin revealed that addition of humic acid to a solution irradiated with visible radiation causes noticeable decomposition, compared to ultra pure water (Wawryniuk et al. 2015). Venlafaxine, regarded to be photostable, reacts with AOS (mainly with the hydroxyl radical, but also with singlet oxygen) and hydrated electrons formed during humic acid solution irradiation (Santoke et al. 2012).

The classical Fenton process (Eqs. 6 and 7) can be combined with UV (photo-Fenton process). Fe^3+^ ions created in the reaction of Fe^2+^ ions with H_2_O_2_ have the tendency to form aqua-complexes, as an effect of hydrolysis. These complexes undergo photolysis under UV and VIS irradiation, producing regenerated Fe^2+^ ions (therefore, irradiation increases regeneration of Fe^2+^) and additional hydroxyl radicals (Eq. 8) (Papoutsakis et al. 2016). Even more hydroxyl radicals are formed during H_2_O_2_ photolysis (Eq. 9). These reactions, as well as direct photolysis of contaminants, make photo-Fenton more effective than the regular Fenton process (Ribeiro et al. 2015; Ammar et al. 2016).

6 $$ {Fe}^{2+}+{\mathrm{H}}_2{\mathrm{O}}_2\to {Fe}^{3+}{OH}^{-}{HO}^{\cdotp } $$

7 $$ {Fe}^{3+}+{\mathrm{H}}_2{\mathrm{O}}_2\to {Fe}^{2+}{HO_2}^{\cdotp }+{\mathrm{H}}^{+} $$

8 $$ {\left[Fe{\left({\mathrm{H}}_2\mathrm{O}\right)}_5(OH)\right]}^{2+}+hv\to {Fe}^{2+}{\mathrm{H}}^{+}{+HO}^{\cdotp } $$

9 $$ {\mathrm{H}}_2{\mathrm{O}}_2\overset{hv}{\to }2{HO}^{\cdotp } $$

The mineralization level of micropollutants during secondary treatment in a wastewater treatment plant reached 97 % after 50 min of photo-Fenton, and drugs such as ciprofloxacin, atenolol, furosemide, or naproxen were completely removed (Klamerth et al. 2013). The main disadvantage of photo-Fenton is the need to maintain a pH lower than 3 to avoid precipitation of ferric hydroxides. The process of acidification and then neutralization is expensive; thus, circumneutral pH modifications of the photo-Fenton process were suggested. Klamerth et al. compared the efficiency of regular photo-Fenton (pH = 3) and two modified versions (neutral pH) of this process: with addition of humic acids and ethylenediamine-*N*,*N′*-disuccinic acid (EDDS) as complexing agents, preventing Fe^3+^ ion precipitation. However, the traditional photo-Fenton process provided the shortest time of treatment; all of the three studied methods demonstrated removal of over 95 % of contaminants. Modification with the use of EDDS was found the most promising (Klamerth et al. 2012). The modified photo-Fenton at near-neutral pH was found effective for removal of azo-dye Trypan blue. Coagulation or precipitation of Fe^3+^ ions did not occur because of low concentration of FeSO_4_ (Dutta et al. 2015). In another study by Klamerth et al., use of ferrioxalate in the photo-Fenton process was studied. Formation of iron-oxalate complexes prevented Fe^3+^ ions from precipitation at near-neutral pH. In addition these complexes are photosensitive, which allows solar radiation to be used (Klamerth et al. 2011).

**Fig. 2:**
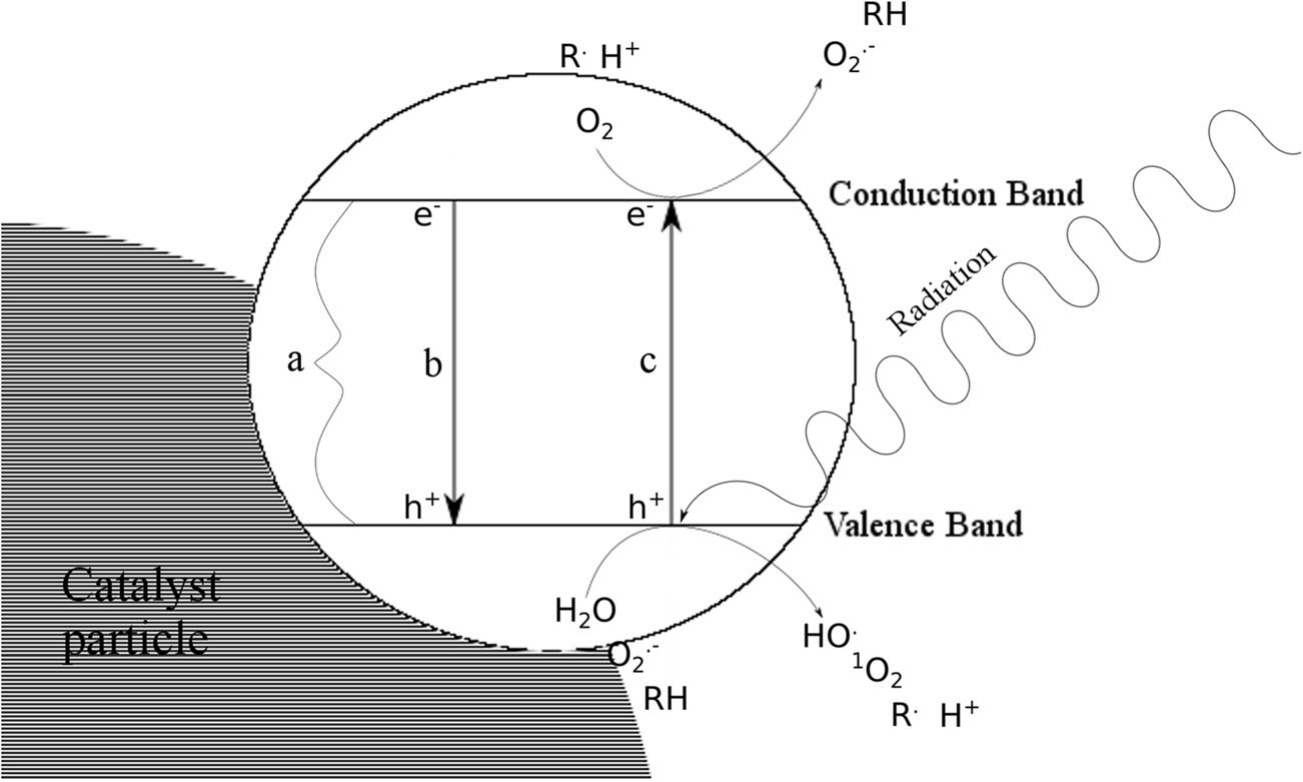
Mechanism of photocatalysis. Band gap (*a*); electron–hole pair recombination (*b*); electron excitation (*c*)

Because of costs, use of photo-Fenton in wastewater treatment plants is limited, despite its high efficiency. Nowadays, most studied methods, taking into account AOPs, are those based on heterogeneous catalysis. Applied catalysts are semiconductors, and their irradiation causes excitation of electrons from valence band to conduction band. This process leads to the formation of an electron–hole pair (Prieto-Rodriguez et al. 2012). Positively charged holes oxidize water to hydroxyl radical and combine with an electron from the donor molecule or oxygen, forming a superoxide radical. The electron–hole pair can also create other AOS (Kabra et al. 2004). Although numerous semiconductors that could be potentially applied as photocatalysts are known, only some of them meet the characteristics of an ideal photocatalyst. Titanium dioxide possesses most of these features: it is highly photostable, photoreactive, non-toxic, chemically and biologically inert, and inexpensive; therefore, it is the most commonly used photocatalyst (Friedmann et al. 2010; Prieto-Rodriguez et al. 2012; Kanakaraju et al. 2014). One of the most important factors is excitation with visible or near-UV light, which depends on a semiconductor bandgap – 3.2 eV in the case of TiO_2_ (Sousa et al. 2013a), which means that the wavelength necessary to promote electron to conduction band is 368 nm (provided by the sunlight spectrum) or less. Processes occurring on an irradiated TiO_2_ particle include formation of a hydroxyl radical, an organic molecule radical, a singlet oxygen, a proton, and a superoxide anion radical (Fig. 2) (Nakata and Fujishima 2012).

The excited state being less stable has a short lifetime, then recombination of an electron and hole takes place and thermal energy is emitted (Kabra et al. 2004).

The main factors influencing photocatalytic properties of TiO_2_ are particle size, crystal composition, band gap, and surface hydroxyl density. A comparison of various TiO_2_ catalysts revealed that Degussa P25 is the highest photoreactive, consisting of 75 % anatase and 25 % rutile with particle size of 20 nm and surface area of 50 m^2^ g^−1^. Although other investigated catalysts consisted of smaller particles (and then larger surface area), their catalytic activity was lower, probably as a consequence of crystal composition (100 % anatase). Rutile, present in P25, has a smaller band gap than anatase, and absorbs photons; then, electrons promoted to the conduction band are transferred to the anatase phase. This results in inhibition of the recombination process (Hurum et al. 2003; Ahmed et al. 2011).

In spite of their proven effectiveness, bare TiO_2_ catalysts have also some disadvantages—a high recombination rate for the electron–hole pairs, a small amount of photons absorbed in the visible region, or difficulty to support titania on some materials. Therefore, many studies on TiO_2_ modification and alternative photocatalysts have been conducted (Calza et al. 2012). The problem of the TiO_2_ wide band gap could be solved by nitrogen doping. The obtained N-doped catalyst is excited by wavelengths of visible light (over 500 nm) (Pereira et al. 2011). The photoactivity of TiO_2_ can be enhanced by assembling it in nanostructures (such as nanofibers, nanotubes, nanorings, nanorods, and similar), mixing with carbon nanotubes, or modifying its structure with conjugated carbon materials (graphite, fullerene, graphene). In this last case, delocalized conjugated π electrons are responsible for rapid photo-induced charge separation and slow charge recombination in the electron-transfer process (Di et al. 2012). Higher photoreactivity of TiO_2_ can be also achieved by mixing titania and activated carbon, which increases surface area, acid–base properties, and the UV spectrum and modifies catalytic behavior, compared to bare TiO_2_ (Araña et al. 2003; Calza et al. 2012). Shao et al. studied photocatalytic properties (degradation of methylene blue under natural sunlight) of vanadium-, iron-, and niobium-doped TiO_2_. The best performance was achieved by application of VT600 photocatalyst (vanadium doped, calcinated at 600 °C) (Shao et al. 2016). TiO_2_ modified with polyoxotungstates outperformed bare P25 titania in degradation of malachite green under UVA radiation (nevertheless, in the case of 4-chlorophenol, P25 was still better) (Rengifo-Herrera et al. 2016). Immobilization of titania on natural materials was also investigated, for instance its loading on volcanic soil (Borges et al. 2016). The obtained catalyst allowed removing 95 % of pollutants from wastewater in 2 h under the visible radiation. The photocatalyst consisting of TiO_2_ immobilized on natural material diatomite possessed attractive physical properties (high porosity and easy separation from water) and high photocatalytic activity (Cherrak et al. 2016). Various compounds are considered as possible alternatives to titanium dioxide, such as binary oxides (ZnO, Cu_2_O, WO_3_, V_2_O_5_, Bi_2_O_3_, Fe(III) oxides and hydroxides, niobium oxides, Ta_2_O_5_, ZrO_2_, CeO_2_, and Ga_2_O_3_), binary sulfides (CdS, ZnS, Bi_2_S_3_, MoS_2_, and Sb_2_S_3_), ternary oxides (vanadates, Bi_2_WO_6_, Bi_2_MO_6_, ZnWO_4_, CdWO_4_, PbWO_4_, Zn_2_SnO_4_, Pb_2_Sn_2_O_6_, indates, gallates, antimonates, bismutates, titanates, ferrites, and niobates), bismuth oxyhalides, indium hydroxides, quaternary oxides, and quaternary oxyhalides (Di et al. 2012). Amongst them, the most studied are photocatalysts based on ZnO. According to studies, photoactivity of ZnO is satisfactory—in some cases, it is slightly less efficient than TiO_2_, but sometimes, photodegradation rates are even higher (Percherancier et al. 1995; Kandavelu et al. 2004) Applicability of zinc oxide is limited, because irradiation causes its photodecomposition. Moreover, ZnO dissolves in strongly basic and acidic solutions (Doménech and Prieto 1986; Calza et al. 2012). Similarly to TiO_2_, ZnO photocatalytic properties can also be enhanced by addition of dopants. Anionic dopants can increase absorption of visible light by catalyst and narrow its band gap. Addition of cationic dopants (transition metals, group I and V elements) significantly enhances photocatalytic properties in comparison to bare ZnO. Promising results were also obtained by rare element doping (Lee et al. 2016). Hierarchically structured nanocomposites are another interesting group of photocatalysts. Such structures possess increased photocatalytic properties because of band alignment, multiple reflections, and scattering of light. Hierarchical photocatalysts can be organized in brunch, brush-like, dendritic, tree-like, urchin-like, flower-like, photonic crystals or macro and mesoporous structures (Padmanaban et al. 2016; Li et al. 2016).

Effectiveness of wastewater treatment methods based on photolysis is often assessed by testing them on large, heterogeneous groups of pharmaceuticals. Kim and Tanaka investigated degradation of 30 personal care products (mainly pharmaceuticals) under UV-C radiation in pure water. The lamp emitting radiation at 185 and 254 nm was more efficient than the lamp emitting only 254 nm. Some of the investigated compounds were highly resistant to this treatment (Kim and Tanaka 2009). Kawabata et al. also studied the treatment method based on direct photolysis (solar, UV-A, UV-B, and UV-C radiation, nine pharmaceuticals). UV-C radiation was the most effective (able to decompose all of the studied drugs), and solar and UV-B less and comparable between each other, and UV-A was the least effective in removal of pharmaceuticals. Toxicity studies showed increase of toxicity in some cases (formation of toxic phototransformation products) (Kawabata et al. 2013). O_3_ treatment combined with application of UV radiation was studied by Kim et al. (30 pharmaceutical products). Addition of UV increased the efficiency of photodegradation in the case of all studied compounds, which was attributed to direct photolysis and formation of hydroxyl radicals (Kim et al. 2011). Comparison of the photodegradation rates (40 pharmaceutical compounds) under LP, MP mercury lamps, and a combination of them with H_2_O_2_ showed that the effectiveness of the LP lamp is lower than that of the MP lamp. Addition of H_2_O_2_ resulted in removal of most of the drugs (however, some of them were resistant to such treatment). Presence of organic matrix (river waters) enhanced photodegradation rates in case of LP and MP lamps alone, but effectiveness of UV/H_2_O_2_ was decreased (hydroxyl radical scavenging) (Wols et al. 2013). Combination of UV radiation with H_2_O_2_ was investigated also by Kim et al. (30 pharmaceuticals and personal care products). Obtained results were similar: while UV alone was insufficient to remove most of the studied compounds, addition of H_2_O_2_ resulted in 90 % degradation of 23 substances after 30 min (Kim et al. 2009). Klamerth et al. compared the regular and modified photo-Fenton methods for removal of over 60 emerging contaminants (including pharmaceuticals). Although conventional photo-Fenton was the most effective method, application of its modified versions also resulted in elimination of over 95 % of contaminants (Klamerth et al. 2012; Klamerth et al. 2012; Klamerth et al. 2013). Application of the photocatalytic method with use of suspended TiO_2_ and solar radiation resulted in complete removal of 19 out of 22 studied pharmaceuticals. *Vibrio fisheri*-based bioassay showed that the applied method did not increase acute toxicity of effluent (Sousa et al. 2012). A study by Prieto-Rodriguez et al. revealed that application of low TiO_2_ concentrations (tens of mg L^−1^) for photocatalytic treatment is insufficient for the degradation of emerging contaminants (mainly pharmaceuticals) (Prieto-Rodriguez et al. 2012). A comparison of photocatalytic treatment methods with the use of TiO_2_ and ZnO by Teixeira et al. (14 pharmaceuticals) revealed that the application of TiO_2_ required six times longer irradiation than ZnO to reach the same level of drug degradation. Nevertheless, ZnO is more problematic because of its potential toxicity (Teixeira et al. 2016). Although the aim of these “multi-drug” studies is to prove if the novel method can be considered as effective against compounds from various chemical groups, as can be seen, they often fail in identification of phototransformation products and assessment of their toxicity.

## Phototoxicity of pharmaceuticals

Phototoxicity, also called photoirritation, the acute reaction occurring after exposure of new chemical entities, belongs to a group of photobiological reactions. Besides phototoxicity, the mentioned group comprises also the following:

- Photoallergy—an immunologically mediated reaction, occurring after multiple exposures of a new chemical entity (photoexcited molecules and proteins form an antigen)
- Photogenotoxicity—possible transformation, caused by visible or UV radiation, of a compound resulting in formation of genotoxic species
- Photocarcinogenity—possible transformation, caused by visible or UV radiation, of a compound resulting in formation of carcinogenic species (Henry et al. 2009; Arimoto-Kobayashi 2014)

Although UV-A radiation is weakly absorbed by proteins and nucleic acids, UV-A is considered one of the causes of skin cancers. This suggests an important role of photosensitizers (also pharmaceuticals), substances absorbing photons followed by molecule excitation (Arimoto-Kobayashi 2014). Two mechanisms of reaction of these species with DNA and proteins are known: type I mechanism, involving one-electron transfer, and type II mechanism which can be divided into two subtypes—major mechanism based on reaction with DNA via singlet oxygen, resulting in formation of 8-oxoguanine, and minor mechanism based on formation of a superoxide anion radical which undergoes disproportionation reaction, giving hydrogen peroxide and reactive oxygen species (IARC 2012). Formation of ROS during photolysis of photosensitizing pharmaceuticals was extensively studied on quinolones. Reaction of a triple state excited molecule results in formation of singlet oxygen and a superoxide anion radical (Eqs. 10–14, *Sens* photosensitizer, *D* electron donor) (Umezawa et al. 1997). When an irradiated molecule reacts with water, hydroxyl radical is formed. Then, further reaction with guanine occurs (Spratt et al. 1999).

10 $$ \mathrm{Sens}{\overset{hv}{\to}}^1{\mathrm{Sens}}^{*}{\to}^3{\mathrm{Sens}}^{*} $$

11 $$ {}^3{\mathrm{Sens}}^{*}+{\mathrm{O}}_2\to \mathrm{Sens}{+}^1{\mathrm{O}}_2 $$

12 $$ {}^3{\mathrm{Sens}}^{*}+{\mathrm{O}}_2\to {\mathrm{Sens}}^{+\cdotp }+{{\mathrm{O}}^{-\cdotp}}_2 $$

13 $$ {}^3{\mathrm{Sens}}^{*}+\mathrm{D}\to {\mathrm{Sens}}^{-\cdotp }+{\mathrm{D}}^{+\cdotp } $$

14 $$ {\mathrm{Sens}}^{-\cdotp }+{\mathrm{O}}_2\to \mathrm{Sens}+{{\mathrm{O}}^{-\cdotp}}_2 $$

Pharmaceuticals representing various therapeutic groups are supposed or known phototoxic compounds. Amongst psychotropics, phenothiazine derivatives are well known for their phototoxic properties. Exposure of the skin to sunlight during therapy with antipsychotics belonging to this group results in phototoxic response and photoallergy. Agranulocytosis, a severe drug side effect, is also linked with photoreactivity of phenothiazine derivatives (Caffieri et al. 2007). Photomutagenic properties of chlorinated phenothiazine derivatives are attributed to induction of UV-A radiation dechlorination followed by formation of the promazyl radical which binds covalently to the DNA molecule (Gocke 1996). Irradiation of chlorpromazine induces formation of photosensitized intermediates which form photoadducts with DNA; researches by Arimoto-Kobayashi and Kochevar confirmed also its photomutagenicity (Kochevar 1981; Arimoto-Kobayashi 2014). Exposure of perphenazine to UV-A radiation results in dechlorination reaction. Under identical conditions, thioridazine gives two major products (endocyclic sulfoxide and endocyclic *N*-oxide with the methylsulfonyl group substituted by a hydroxyl group), similarly to fluphenazine (*N*-piperazine oxide and the trifluoromethyl group substituted by a carboxyl group). Toxicity of these photoproducts, as well as parent compounds, was assessed using hemolysis and 3T3 fibroblast viability assays (Miolo et al. 2006). The trifluoromethyl group of fluphenazine turned out to be labile and susceptible to nucleophilic attack when a molecule is excited by UV radiation. This results in formation of various products whose structures depend on the applied solvent (e.g., carboxylic acid in water or methyl ester in aqueous methanol). When the drug is irradiated in the presence of amino acids at pH = 7.4, it covalently binds to the α-amino group and to other nucleophilic residues (of course only in the case of amino acids possessing such groups—lysine, tyrosine, or cysteine). In vivo, this binding to amino acids of proteins is supposed to cause formation of antibodies and, as a consequence, destruction of granulocytes and agranulocytosis (Caffieri et al. 2007). Studies on photochemistry of levomepromazine showed that phototoxicity of this drug is based on the type II mechanism—formation of singlet oxygen which is able to react with histidine followed by formation of *trans*-annular peroxide (Vargas et al. 2003). Irradiation of trifluoperazine gives, besides other products typical for phenothiazine derivatives, 3-trifluoromethyldibenzo[b,d]thiophene sulfoxide (Abdel-Moety et al. 1996). Cyamemazine, a drug inducing strong skin phototoxicity, undergoes rapid photolysis when it is exposed to UV-A radiation. The main photoproduct (*N*-oxide formed on a side chain) can act as a type I and type II photosensitizer. Formation of this photoproduct involves oxygen and ROS (mainly superoxide anion radical, singlet oxygen to a lesser extent) formed during the photodegradation process (Morlière et al. 2004).

Another group of phototoxic psychotropic drugs are tricyclic antidepressants (TCA) which exhibit susceptibility to UV radiation (Székely et al. 2010; Székely et al. 2011a). A study on imipramine, clomipramine, amitriptyline, nortriptyline, and doxepin demonstrated the phototoxic effect of intermediate photodecomposition products on erythrocytes (cell lysis) (Székely et al. 2011b). UV irradiation of clomipramine results in formation of four photoproducts (imipramine, OH-imipramine, desmethyl clomipramine, and OH-imipramine N-oxide). More photoproducts are formed as a result of doxepin photodegradation, but only two of them were identified (OH-doxepin and doxepin N-oxide) (Székely et al. 2011a). Irradiation of imipramine and amitriptyline induces production of singlet oxygen. Photodecomposition of the latter pharmaceutical involves creation of a significant amount of superoxide anion. Both drugs are phototoxic, causing cell death in the 3T3 test (Viola et al. 2000).

Carbamazepine is also well known for its phototoxicity. Eczematous reaction caused by this drug was reported after skin exposure to light. Study by Teuri and Tagami proved that this kind of photo-induced reaction is a consequence of UV-A irradiation (Terui and Tagami 1989; Onoue and Tsuda 2006).

Pharmaceuticals such as fluoroquinolones (e.g., ciprofloxacin), tetracyclines (e.g., tetracycline), sulfonamides (e.g., sulfamethoxazole), thiazide diuretics (e.g., chlorothiazide), NSAIDs (e.g., ketoprofen), and sulfonylurea-class anti-diabetics (Arimoto-Kobayashi 2014) are also reported as phototoxic substances.

Detection of reactive oxygen species, products of photosensitization reactions, could be used to assess the photoreactivity of an irradiated substance. Although direct observation of phosphorescence of singlet oxygen is preferred, its application is limited. Therefore, frequently applied are indirect methods such as bleaching of *N*,*N*-dimethyl-*p*-nitrosodimethylaniline monitoring, using imidazole as a selective acceptor of singlet oxygen. Assay of superoxide anion radical can be based on photosensitized reduction of nitroblue tetrazolium with formation of nitroblue formazan as an intermediate (Kleinman 2013).

Toxicity of phototransformation products may be assessed using in vitro studies. Commonly used are assays on *V. fischeri* (based on inhibition of the luminescence emitted by bacteria) and *D. magna* (Trovó et al. 2009; Vasconcelos et al. 2009). Cytotoxicity to human cells can be measured using water-soluble tetrazolium assay and neutral red uptake assay (Bergheim et al. 2014). To detect genotoxic potential, SOS Chromotest, Ames test (Fatta-Kassinos et al. 2011b), and fluorimetric detection of DNA unwinding test (Bergheim et al. 2014) are used.

## Photodegradation studies on psychotropic drugs

**Fig. 3:**
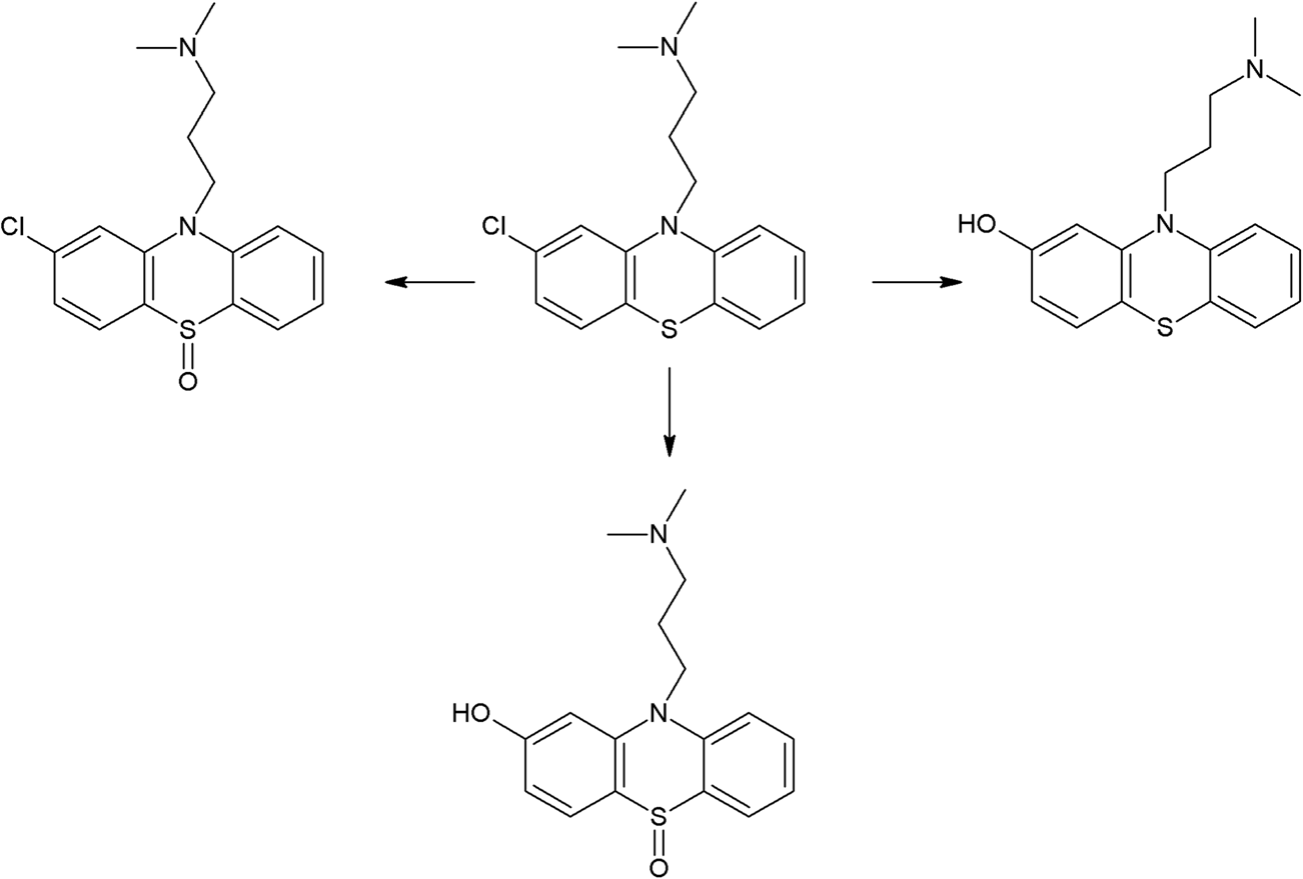
Chlorpromazine and its three main photoproducts after 4 h irradiation with xenon lamp (Trautwein and Kümmerer 2012)

On the photodegradation aspect, phenothiazine derivatives are the most studied group of antipsychotics. In the case of chlorpromazine, very extensive photodegradation with use of a xenon lamp was reported by Trautwein and Kümmerer (2012). In this study, after 4 h of irradiation, almost complete degradation of chlorpromazine was observed and 58 photodegradation products were formed (Fig. 3).

According to another study, the photodegradation pathway of chlorpromazine depends on the reaction medium. In aqueous solution in the presence of oxygen, chlorpromazine sulfoxide and nitroxide are formed. Irradiation of deoxygenated aqueous solution yields to formation of 2-hydroxychlorpromazine and the corresponding dimers. In the presence of nucleophilic organic solvents, chlorine is substituted by a solvent molecule (Kochevar 1981).

The trifluoromethyl group in the fluphenazine molecule turned out to be photolabile, which results in its hydrolysis and then substitution by a carboxylic group (in aqueous medium). Irradiation of aqueous methanol, ethanol, and ammonia results in formation of methyl ester, ethyl ester, and amide respectively. Formation of another photoproduct—piperazine N-oxide, is not solvent dependent (Miolo et al. 2006; Caffieri et al. 2007).

**Fig. 4:**
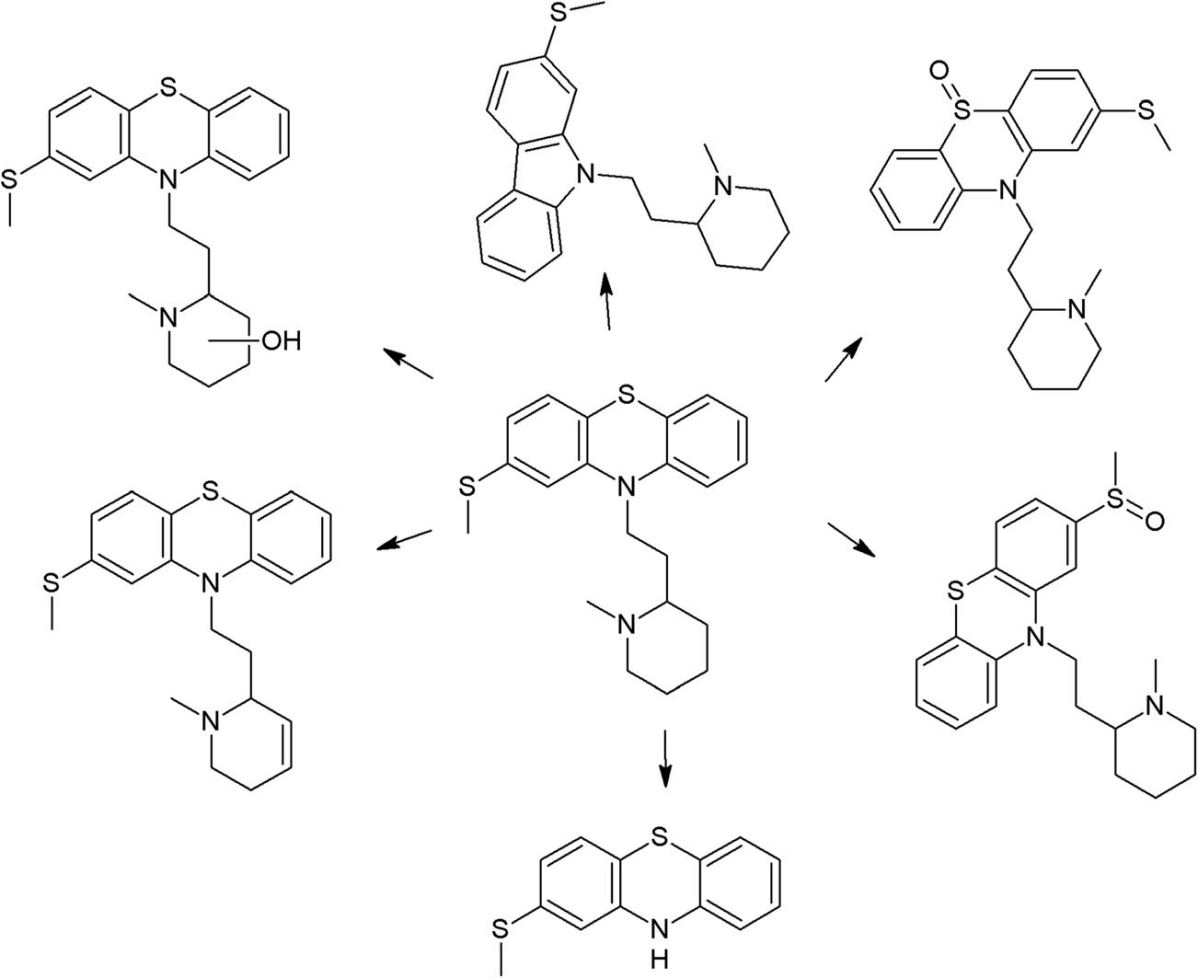
Thioridazine and its selected photoproducts (Wilde et al. 2016)

Numerous photoproducts were found after irradiation of thioridazine dissolved in pure water with the use of a xenon lamp (Fig. 4). Two main were identified as 5-sulfoxide and 2-sulfoxide. Measured half-life times were 5.84 and 8.08 min for initial concentrations 50 and 0.5 mg L^−1^ respectively. Toxicity (measured with the use of *V. fisheri*) was decreasing in parallel with thioridazine phototransformation. QSAR analysis revealed that carbazole derivatives formed during phototreatment may possess mutagenic or genotoxic properties (Wilde et al. 2016).

The main photoreaction pathway in the case of thioxanthene derivatives is photoisomerization. Kopelent-Frank and Mittlböck reported that the *E*-chlorprothixene was formed after *Z*-chlorprothixene irradiation. Oxidation reaction was also observed, and 2-chlorthioxanthone was also found (Kopelent-Frank and Mittlböck 1996).

**Fig. 5:**
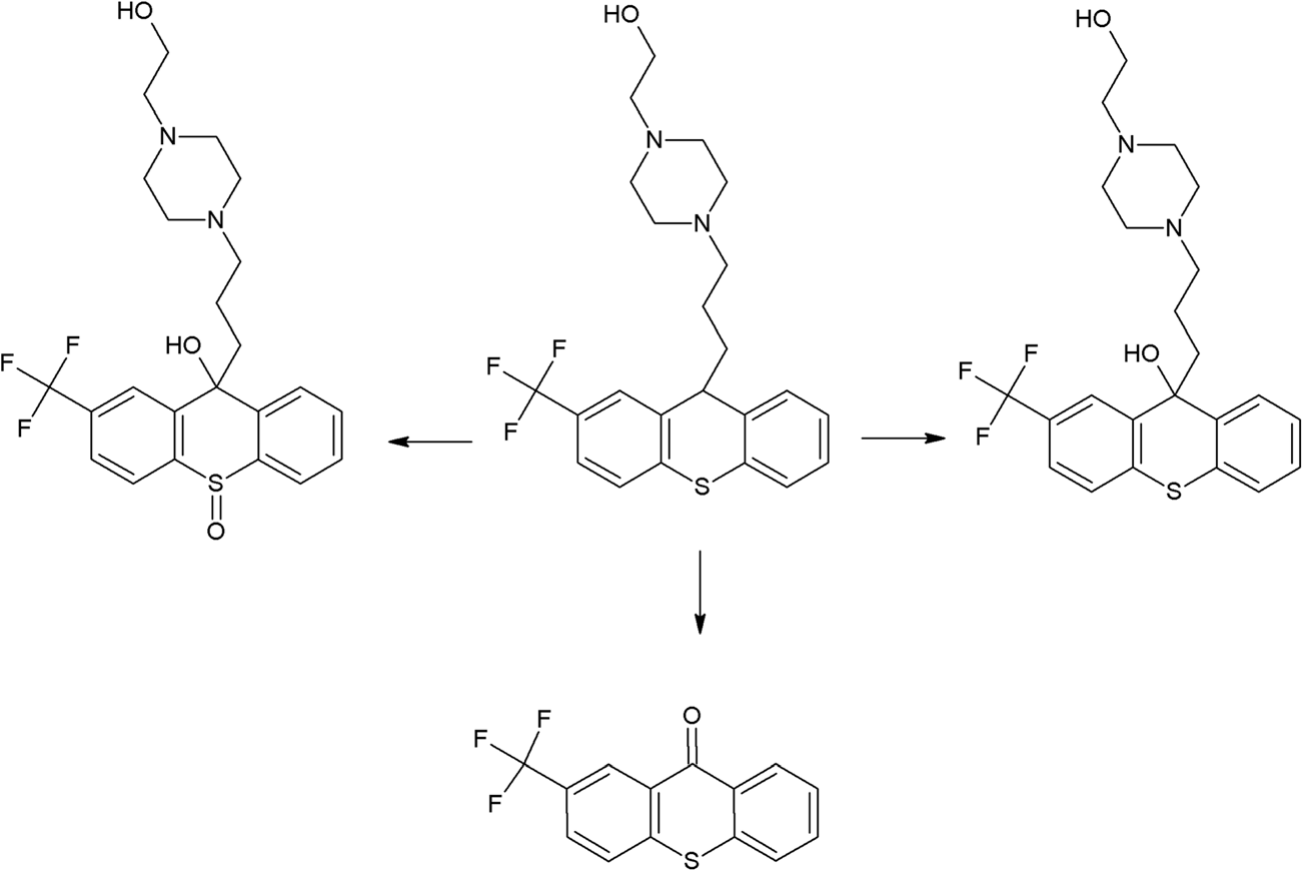
Structures of flupentixol and its three main photoproducts ((Maquille et al. 2010)

Irradiation of flupentixol with UV-C results in formation of 10 photoproducts as a consequence of oxidation (formation of sulfoxide or ketone), hydroxylation of a double bond, cleavage or elimination of a piperazine ring, or elimination of the whole side chain (Fig. 5) (Maquille et al. 2010).

A stability-indicating study on butyrophenones showed that droperidol and benperidol are susceptible to UV-C radiation, while haloperidol is photostable (Sanli et al. 2013). No relevant researches on photostability of diphenylbutylpiperidine derivatives have been done—only exposure of pimozide on direct sunlight for 5 h was studied (Manjula and Ravi 2013).

Nowadays, much more photodegradation studies have been performed on atypical antipsychotics, but in the case of some drugs, conclusions are incoherent. As an example, at least three studies proved that olanzapine in aqueous solutions is a photostable molecule, even under UV-C irradiation. Krishnaiah et al. investigated photolysis of olanzapine in buffered aqueous solution under 254 nm irradiation. After 10 days, no degradation was observed (Krishnaiah et al. 2011). Regulska and Karpińska studied direct (254 and 366 nm, aqueous solution) and indirect photolysis (river waters and TiO_2_ photocatalysis, simulated solar radiation) of olanzapine. The compound was resistant to UV irradiation alone, but turned out to be susceptible to indirect photolysis. A similar study (almost the same conditions of photolysis and findings) was done by Karpińska et al. (2012; Regulska and Karpińska 2012). On the contrary, Pathak and Rajput reported that olanzapine (in neutral, acidic, and basic solutions) undergoes photodegradation under irradiation in a photostability chamber for 15 days (Pathak and Rajput 2009). Taking into account similar conditions of photolysis experiments, explanation of differences between these studies is problematic. Photostability of olanzapine under UV-C irradiation in solid state was also investigated—no signs of degradation were noticed after 10 days (Shah et al. 2008).

Ziprasidone was also found as a photolabile atypical antipsychotic. According to Żakowiecki and Cal, the free base of ziprasidone underwent mild photolysis under UV–VIS irradiation (irradiation equaled 1.0–1.2 W cm^−2^), while its hydrochloride was stable (Zakowiecki and Cal 2012). One of the suggested photoreactions for this drug is benzisothiazole ring isomerization to isobenzothiazole (photodegradation conditions of this study were not specified) (Sharp et al. 2003). On the other hand, under UV-C (lamp power = 6 W) irradiation of ziprasidone for 96 h, 12 photoproducts were formed as a consequence of an indole ring cleavage or dehydrogenation, dechlorination, dehydrogenation of aliphatic chain or its cleavage. Under UV-A (lamp power = 1.3 W) irradiation, only one photoproduct was formed (Skibiński 2012a]). It should be also noticed that some researches report that ziprasidone is a photostable compound even after 7 days of irradiation (ICH conditions) (Singh et al. 2007). Contradictory findings of these studies may be attributed to application of different radiation sources (wavelengths and source power) and time of experiments.

**Fig. 6:**
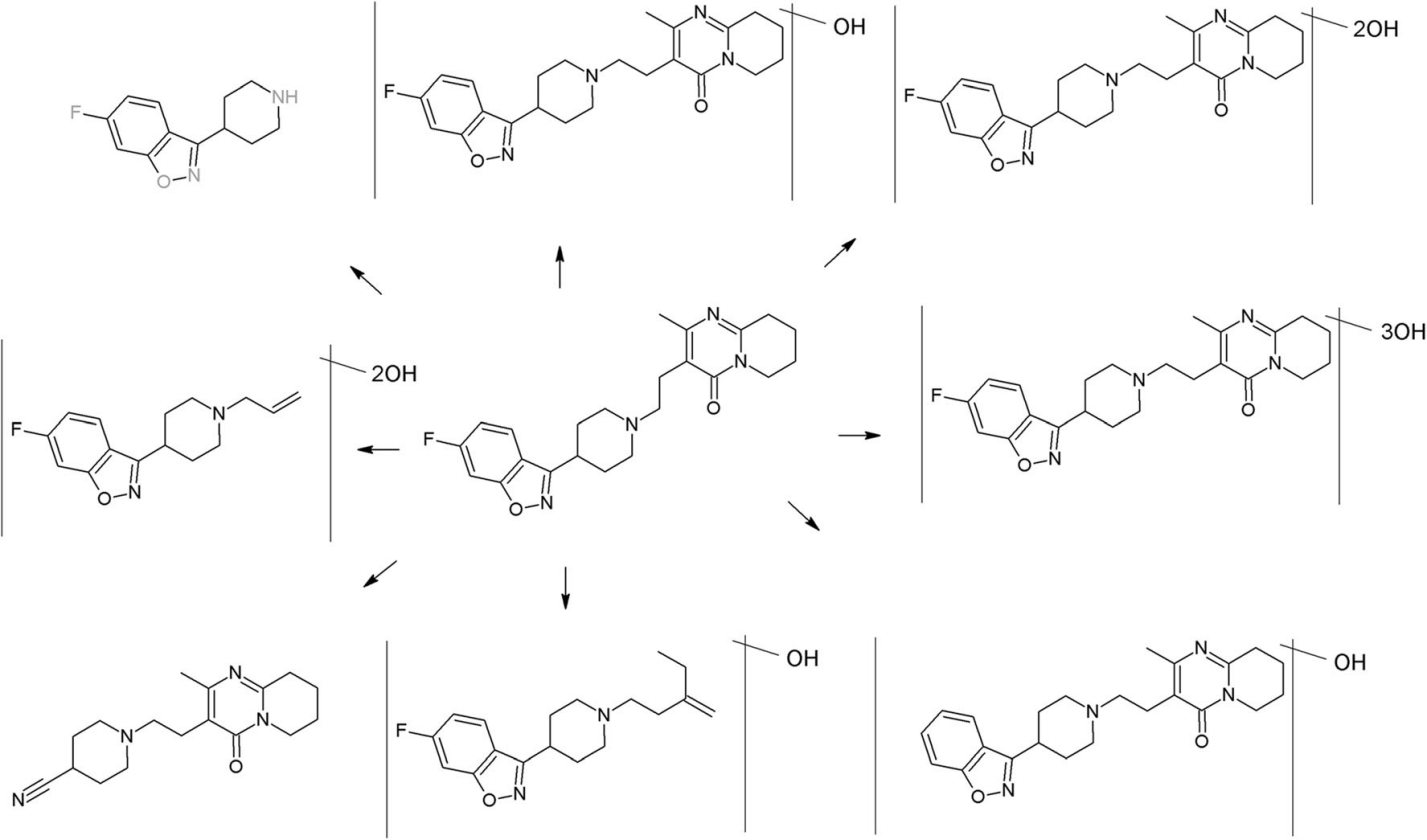
Risperidone and its selected photoproducts (Calza et al. 2016)

Thirty-four photoproducts were detected and identified in photocatalytic degradation of risperidone with the use of reduced graphene–TiO_2_ catalyst (xenon lamp). The use of bare P25 resulted in formation of only 20 products (selected products are showed in Fig. 6). Toxicity of intermediates was higher than that of the parent molecule (assessment with the use of Microtox bioassay). An experiment was conducted in distilled water and surface water. Application of modified photocatalyst gave better effects than bare TiO_2_ (faster mineralization and reduction of toxicity) (Calza et al. 2016).

**Fig. 7:**
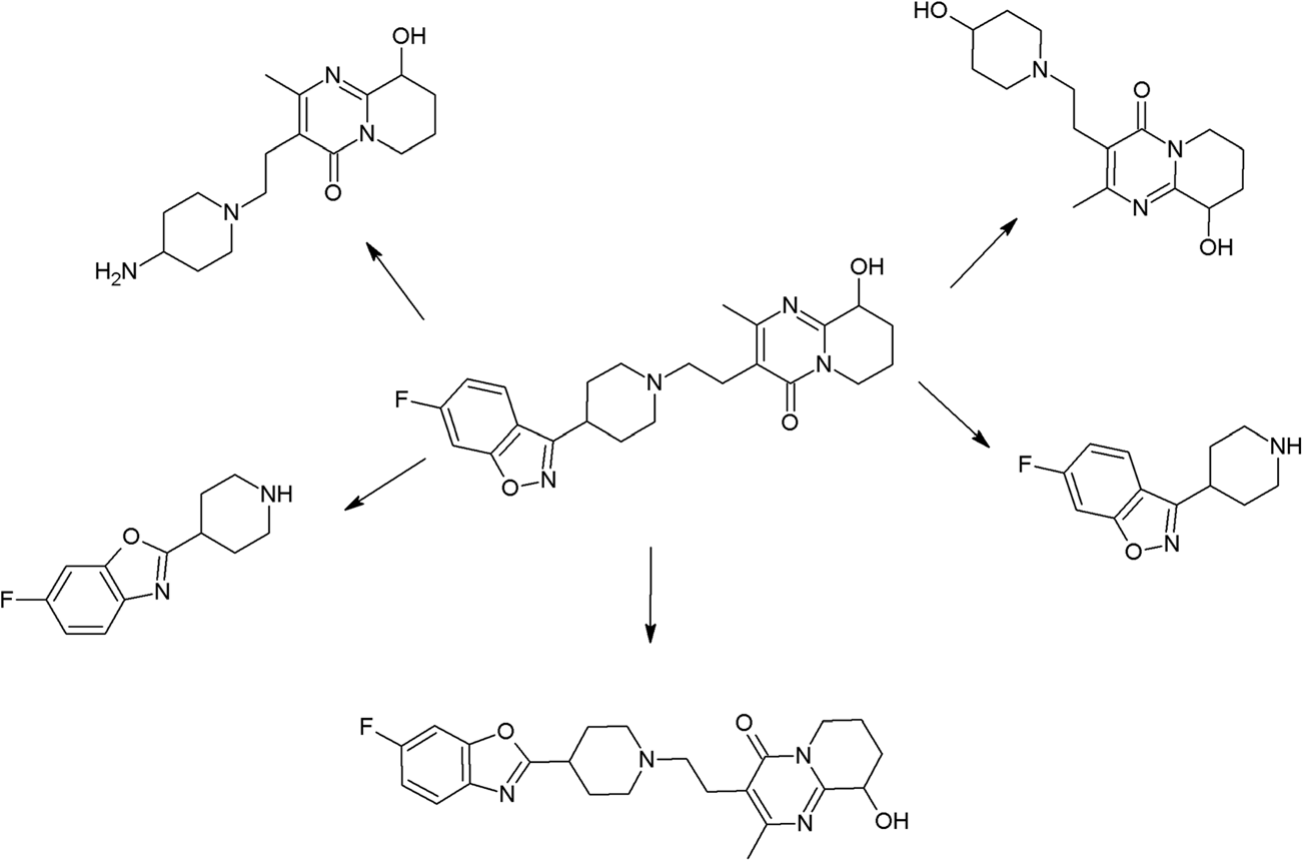
Paliperidone and its photoproducts (Skibiński et al. 2016)

The number of phototransformation products of paliperidone could be dependent on the applied wavelength. Irradiation of methanolic and aqueous solutions with UV-A radiation resulted in formation of only two photoproducts, while with UV-C—five (Fig. 7). A kinetic study showed that under UV-A irradiation paliperidone is much less stable than under UV-C (half-lives 0.14 and 825.12 h respectively). According to ECOSAR software, toxicity of three degradation products was close to that of the parent molecule. LC_50_ values for two products were considerably higher than that of paliperidone (Skibiński et al. 2016).

**Fig. 8:**
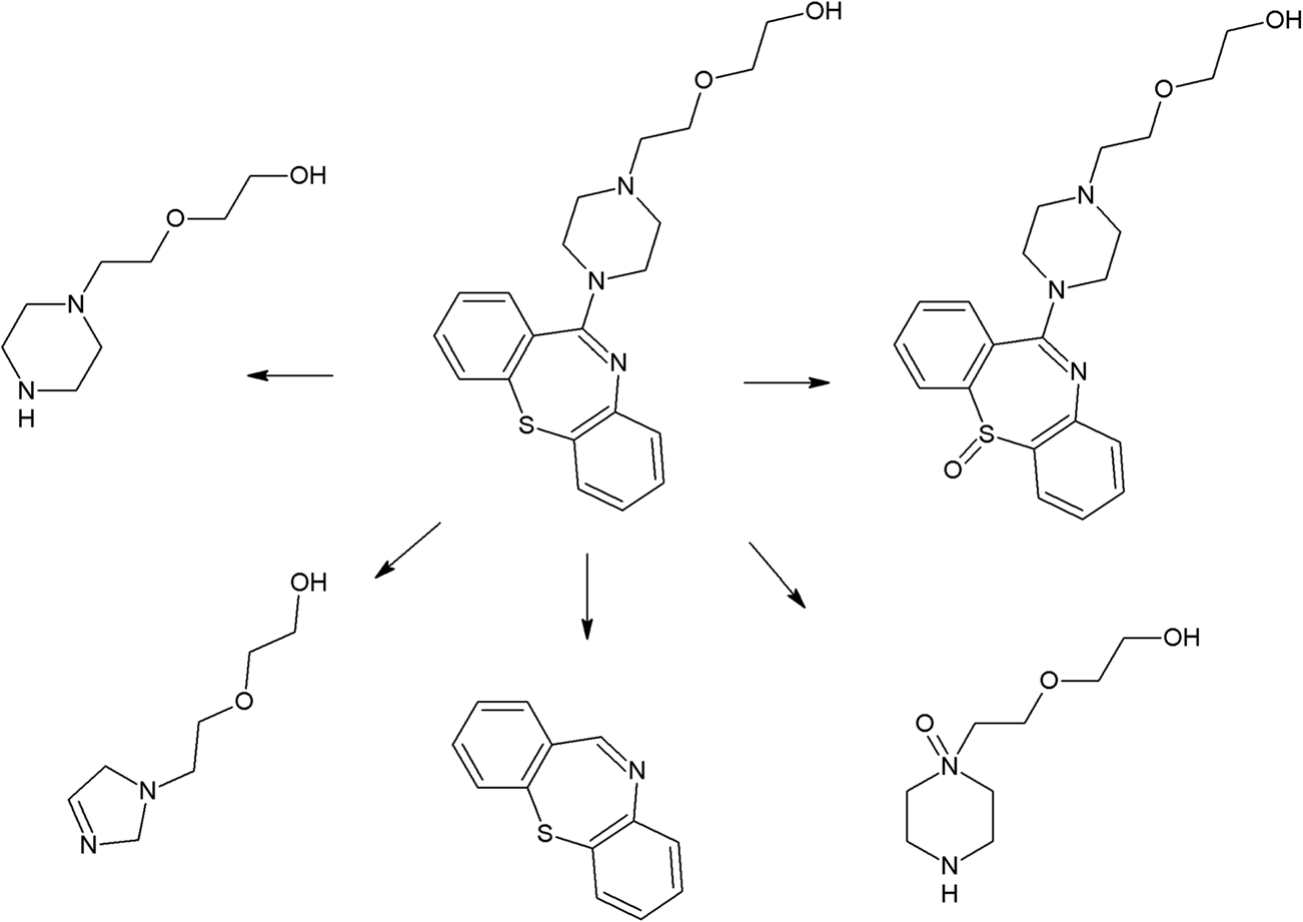
Quetiapine and its photoproducts after irradiation with UV-C radiation (Skibiński 2012b)

Another atypical antipsychotic drug, quetiapine, was identified as photostable under ICH conditions (Soma et al. 2009); however, under UV-C irradiation, contradictory reports were presented. In one paper, quetiapine in methanolic solution was found as a photostable compound under UV-C (254 nm) irradiation (Trivedi and Patel 2011), but at the same time in the above conditions, sensible photodegradation was observed (also methanolic solution, irradiance level = 390 μW cm^−2^, wavelength = 254 nm, irradiance time = 6 h), and 5 photoproducts were identified (Fig. 8) (Skibiński 2012b). The discussion on the differences between these findings is problematic, as an accurate description of photolytic conditions in the first study was not provided.

**Fig. 9:**
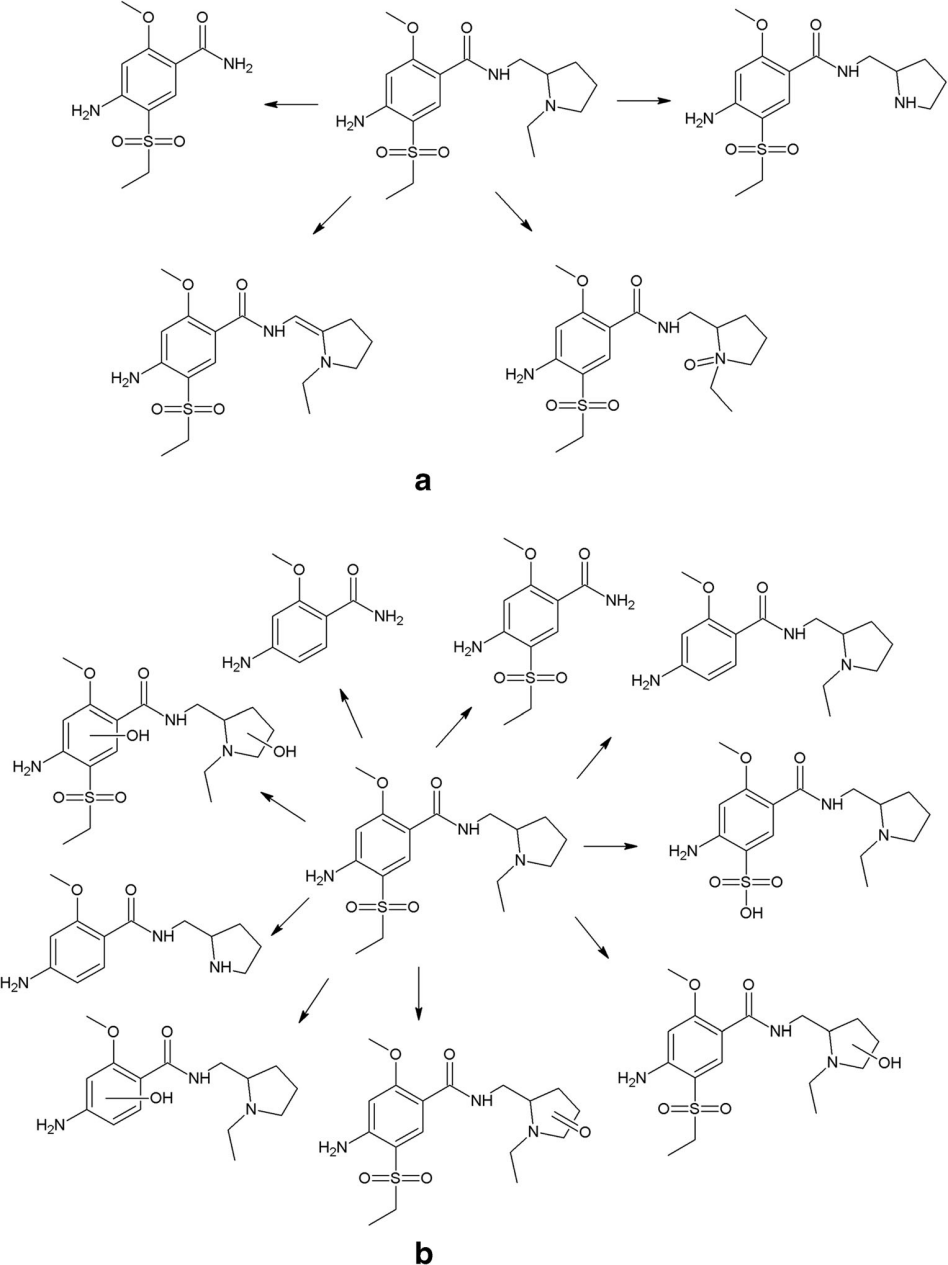
Amisulpride and products of its photodegradation with UV-A radiation (**a**) (Skibiński 2011) and with solar radiation (**b**) (Gros et al. 2015)

A benzamide derivative, amisulpride is reported as a photolabile compound under UV-A, as well as under simulated solar radiation. Its irradiation with 365 nm UV in methanolic solution led to formation of four degradation products (Skibiński 2011) (Fig. 9A). Gros et al. studied photodegradation of amisulpride in ultrapure water and two wastewater samples under simulated solar radiation. A comparison of reaction kinetics revealed that photolysis in ultrapure water resulted in a higher degradation rate than in wastewater. Nine photoproducts were detected (Fig. 9B)—all of them were formed in the direct photolysis experiment, eight as an effect of indirect photolysis (product with two hydroxyl groups was not detected). Only one of the products was identical to those formed in the UV-A photolysis experiment (fragment with detached (1-ethylpyrrolidin-2-yl)ethyl group) (Gros et al. 2015).

**Fig. 10:**
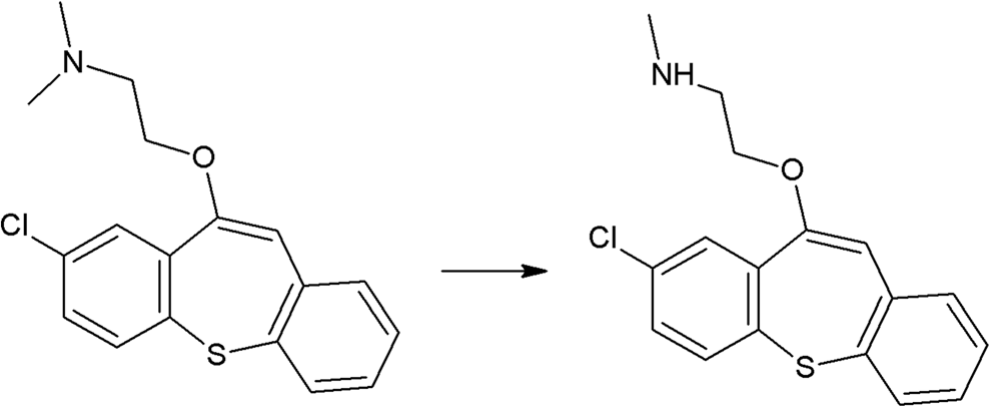
Zotepine and its photoproduct (Talluri et al. 2014)

It is worth noting that irradiation of drug substances in solid state is sometimes an insufficient procedure. In such cases, a forced photodegradation study in solvent medium is recommended, for example, a photodegradation study of zotepine showed its stability under cool white fluorescence lamp radiation in solid state (0.1 % degradation after 72 h), while irradiation of zotepine solution led to rapid decomposition—25 % within 3 h with formation of the main degradation product (Talluri et al. 2014) (Fig. 10).

The literature data concerning the photodegradation of antipsychotic drugs are presented in Table 2.

**Table 2:** Photodegradation studies on antipsychotics

Compound	Medium	Irradiation source	Analysis method	Toxicity assessment	Photoproducts/results	Ref.
Chlorpromazine	Water	Xenon 300–800 nm	HPLC–MS	CBT, MRT, ANAD tests	57 (29 identified)	(Maquille et al. 2010)
	MetOH; EtOH; 2-Pr-OH	Hg lamp (313 nm)	GC–MS ^1^H NMR ^13^C NMR	None	Alkoxide derivatives	(García et al. 2009)
	Water–MetOH	Natural sunlight; 254 nm; 366 nm	HPLC	None	None	(Prohotsky et al. 2014)
Fluphenazine	Water; Various solvents	UV-A	HPLC–MS TLC NMR	Assessment of binding to proteins	7 identified	(Caffieri et al. 2007)
	Water	UV-A	TLC IR NMR MS	Photohemolysis 3T3 fibroblasts viability	2 identified	(Miolo et al. 2006)
	Water buffers	UV-C	EPR EAS SAXS	None	Oxidized products (sulfoxides)	(Rodrigues et al. 2006)
	Water	Daylight; 254 nm	Spectrofluorimetric	None	None (photostable under daylight, photolabilie under UV-C)	(Belal et al. 2008)
Perphenazine	Water	UV-A	TLC IR NMR MS	Photohemolysis 3T3 fibroblasts viability	1 identified	(Miolo et al. 2006)
	PG	Cool white light	HPLC MS	None	2 identified	(Li et al. 2005)
Thioridazine	Water	UV-A	TLC IR NMR MS	Photohemolysis 3T3 fibroblasts viability	2 identified	(Miolo et al. 2006)
	Water buffers	UV-C	EAS EPR SAXS	None	Oxidized products (sulfoxides)	(Rodrigues et al. 2006)
	Water	Xenon lamp	UPLC–MS–LTQ–Orbitrap	CBT, MRT, LBT tests; QSAR	Numerous identified (two main—5-sulfoxide and 2-sulfoxide)	(Wilde et al. 2016)
Flupentixol	Water	UV-C	UHPLC–MS	None	9 identified	(Maquille et al. 2010)
Perazine	MetOH; EtOH; 1-Pr-OH 2-Pr-OH t-ButOH	Hg lamp (313 nm)	GC–MS ^1^H NMR ^13^C NMR	None	Alkoxide derivatives	(García et al. 2009)
Prochloperazine	SS	UV-A	HPLC NMR	None	Over 50, 4 identified (main—perazine)	(Lew et al. 2011)
Trifluoperazine	Water	UV-C; daylight	TLC MS Spectroph.	None	Over 10 (sulfoxide and dibenzotiophene derivative)	(Abdel-Moety et al. 1996)
	Water buffers	UV-C	EAS EPR SAXS	None	Oxidized products (sulfoxides)	(Rodrigues et al. 2006)
	MetOH	UV; White light	HPLC-PDA	None	None (practically photostable)	(Shetti and Venkatachalam 2010)
	SS	Natural sunlight UV	HPLC	None	None	(Subbareddy and Divakar 2016)
Dixyrazine	Dextrose in water	ID 65 Filter; daylight	HPLC	None	9 products (mainly oxidized)	(Kopelent-Frank and Mittlböck 1996)
Chloprothixene	Dextrose in water	ID 65 Filter; daylight	HPLC	None	3 products (mainly oxidized)	(Kopelent-Frank and Mittlböck 1996)
Levomepromazine	MetOH	UV-B	MS NMR Spectroph.	Hemolysis of RBC, Peroxidation of linoleic acid	Sulfoxide	(Vargas et al. 2003)
	River water	300–800 nm; 365 nm	HPLC GC–MS	None	Sulfoxide	(Karpińska et al. 2012)
	Water	Fluorescent light	HPLC	None	None	(Fernandez-Campos et al. 2013)
Cyamemazine	Water	Hg lamp (365 nm); Laser flash photolysis	HPLC MS ^1^H NMR ^13^C NMR	None	Sulfoxide N-oxide	(Morlière et al. 2004)
Haloperidol	MetOH	Natural sunlight; UV	HPTLC	None	1 detected	(Mennickent et al. 2008)
	MetOH	Daylight	HPLC	None	Several detected	(Driouich et al. 2001)
	SS	UV-C	HPLC	None	None (photostable)	(Sanli et al. 2013)
Droperidol Benperidol					None	(Sanli et al. 2013)
Pimozide	MetOH	Direct sunlight	HPTLC	None	None (photostable)	(Manjula and Ravi 2013)
Sertindole	MetOH	UV	TLC HPLC GC–MS IR	None	None (photostable)	(El-Ragehy et al. 2014)
Olanzapine	ACN–Water	UV-C	UHPLC–MS	None	2 identified	(Krishnaiah et al. 2011)
	MetOH	UV-C	HPTLC	None	Photostable	(Shah et al. 2008)
	Water; HCl; NaOH; SS	N/A	HPLC	None	5 major photoproducts	(Pathak and Rajput 2009)
	Water; River water; TiO_2_	300–800 nm; 254 nm; 365 nm	HPLC	None	Susceptible to photocatalysis	(Regulska and Karpińska 2012)
	Water; River waters	300–800 nm; 365 nm	HPLC GC–MS	None	Degradation occurs in the presence of river matrix	(Karpińska et al. 2012)
Ziprasidone	Water	UV–VIS	UHPLC	None	Photodegradation observed only in the case of free base	(Zakowiecki and Cal 2012)
	MetOH	UV-C; UV-A	UHPLC–MS	None	1 photoproduct (UV-A); 12 photoproducts (UV-C)	(Skibiński 2012a)
	MetOH	UV	HPLC	None	None	(Ramesh et al. 2012)
	Water–ACN	ICH	HPLC	None	None (photostable)	(Zakowiecki and Cal 2012)
	MetOH	254 nm	Spectrofluorimetric	None	1 detected	
Quetiapine	MetOH	UV-C	UHPLC–MS/MS	None	5 identified	(Skibiński 2012b)
	Buffer; MetOH–ACN	N/A	HPLC	None	None (photostable)	(Soma et al. 2009)
	Water	UV	HPLC	None	None (photostable)	(Korrapolu et al. 2012)
	Water–ACN	ICH; 254 nm	HPLC–DAD	None	None (photostable)	(Narendra et al. 2011)
	SS		HPLC–PDA	None	None (photostable)	(Kumar et al. 2013)
Levosulpiride	ACN-buffer	UV	HPLC	None	None (photostable)	(Pal et al. 2016)
Amisulpride	MetOH	UV-A	UHPLC–MS/MS	None	4 identified	(Skibiński 2011)
	Water; WW	Simulated solar radiation	UHPLC – MS	*Vibrio fischeri*	9 identified	(Gros et al. 2015)
	SS	Natural sunlight	UPLC–PDA	None	None (mild photodegradation)	(Dabhi et al. 2013)
Zotepine	MetOH–water	N/A	HPLC–MS	None	1 identified	(Talluri et al. 2014)
Clozapine	SS	300–800 nm	HPLC	None	None (photostable)	(Perks et al. 2012)
Risperidone	SS	ICH	HPLC	None	None (photostable)	(Svirskis et al. 2011)
	Water and surface water + reduced graphene-TiO_2_	Xenon lamp	HPLC–MS–LTQ–Orbitrap IC TOC analyzer	Microtox (*V. fisheri*)	34 (reduced graphene–TiO_2_) and 20 (P25 TiO_2_) identified	(Calza et al. 2016)
Aripiprazole	Water; MetOH; TFA	UV-C	HPLC	None	None (photostable)	(Pai and Dubhashi 2013)
	Water; ACN; TFA	UV-C	HPLC	None	None (photostable)	(Srinivas et al. 2008)
	ACN; Water; buffers	ICH	HPLC	None	None (photostable)	(Narayana and Chandrasekhar 2012)
Paliperidone	Water; TiO_2_; Surfactants	Natural sunlight	HPLC	None	1 photoproduct (N-oxide)	(Marothu et al. 2015)
	ACN–MetOH	ICH	HPLC–PDA	None	None (photostable)	(Bindu et al. 2012)
	ACN-buffer	UV	HPLC–PDA	None	None (photostable)	(Sherje and Londhe 2015)
	MetOH; Water	UV-A; UV-C	UPLC–MS/MS	Computational toxicity assessment (ECOSAR)	5 (UV-C) and 2 (UV-A) identified	(Skibiński et al. 2016)
Asenapine	MetOH	Natural sunlight	HPLC–PDA	None	None (photostable)	(Chhalotiya et al. 2012)
	MetOH	UV	HPTLC	None	None (photostable)	(Patel et al. 2015)
Lurasidone	SS; Water–MetOH	ICH	HPLC–MS/MS	None	None (photostable)	(Kumar Talluri et al. 2015)

**Fig. 11:**
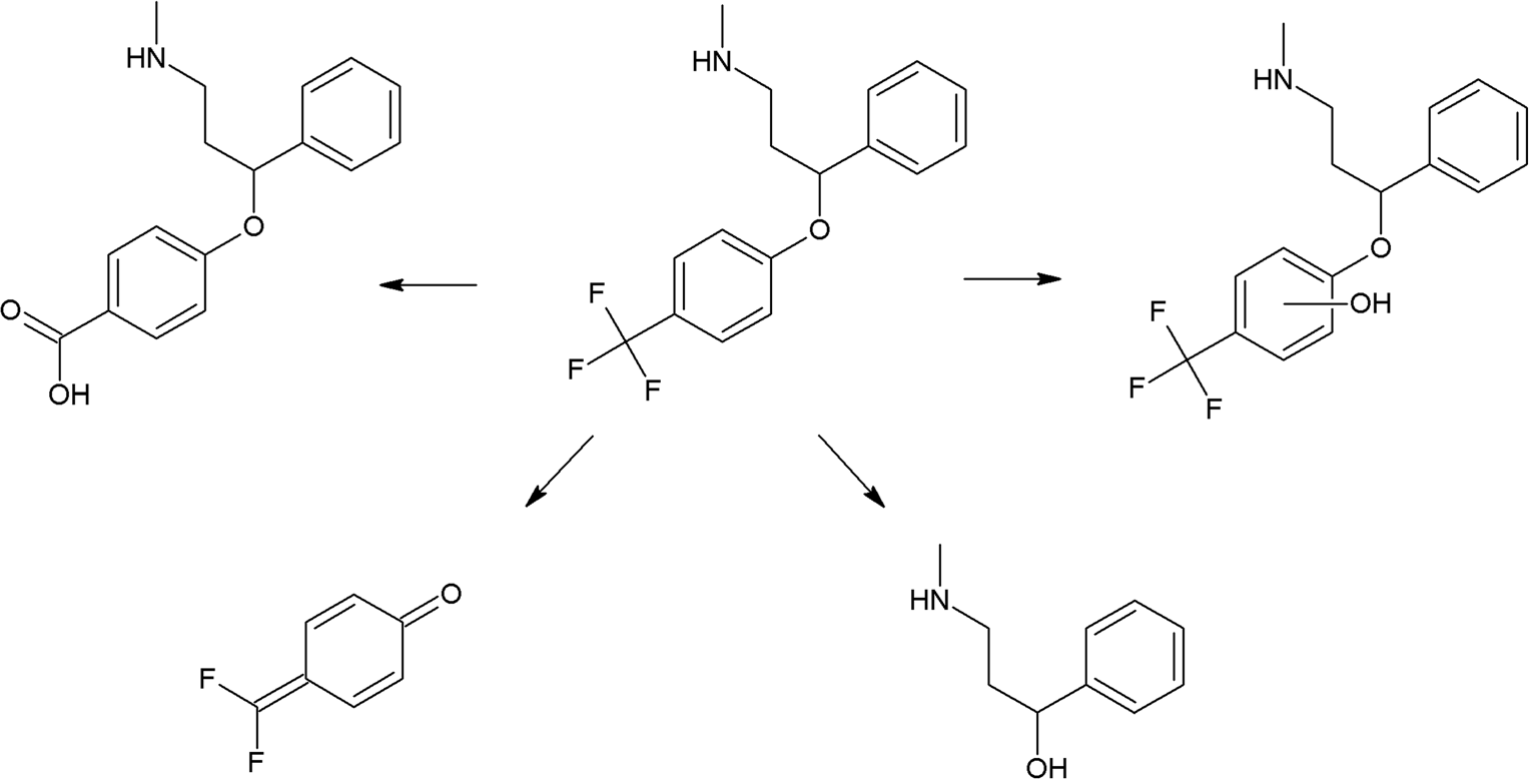
Fluoxetine and products of its direct and indirect (hydroxylation of a phenyl group) photodegradation (Lam et al. 2005)

Amongst antidepressants, the most studied drug is a well-known SSRI agent—fluoxetine which undergoes direct as well as indirect photodegradation. A faster rate of degradation in the presence of DOM or photocatalyst indicates that indirect photolysis plays a major role (Lam et al. 2005; da Silva et al. 2015). However, in the study performed by Yu et al. (2015), fluoxetine was classified as a photo-susceptible compound with minor degradation by HO^·^ radical, because no significant increase of the degradation rate was observed after addition of hydrogen peroxide. One of the photoproducts—consequence of defluorination—proves this type of photoreaction is common for trifluoromethyl compounds (in the case of fluphenazine). Products of direct and indirect photodegradation are shown in Fig. 11. In the case of fluoxetine, results of photodegradation experiments are sometimes ambiguous. Although most of studies confirmed its susceptibility to even simulated solar radiation, Shah et al. stated that fluoxetine, in solid state however, remained irradiation with 254 nm within 10 days (Shah et al. 2008).

**Fig. 12:**
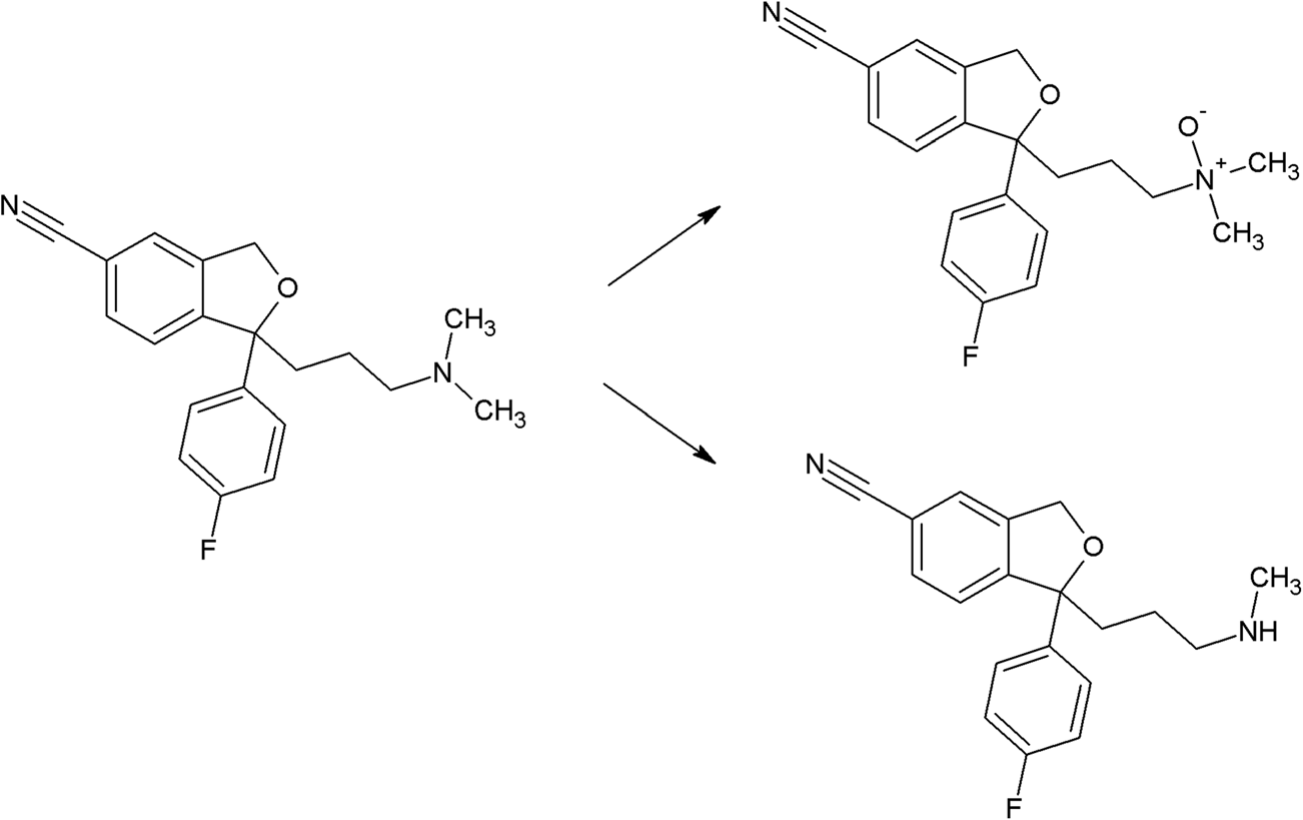
Citalopram and its photoproducts (Kwon and Armbrust 2005a)

**Fig. 13:**
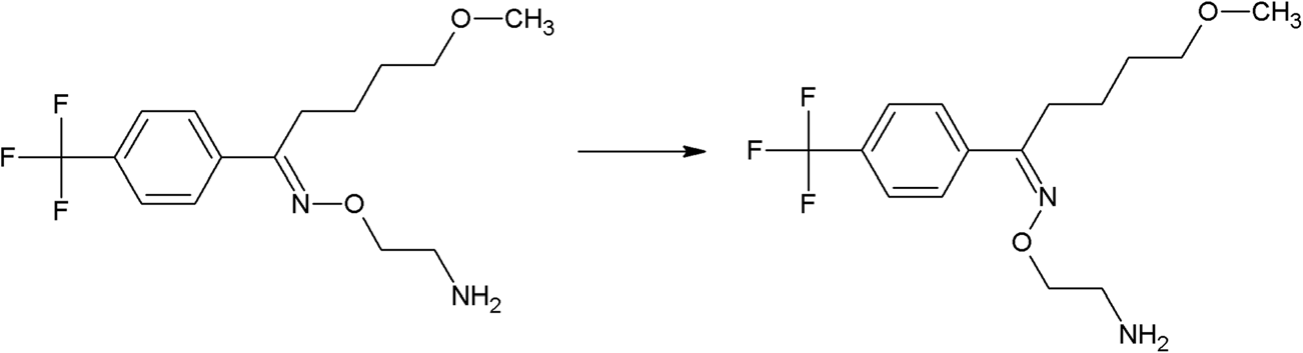
Fluvoxamine and product of its photo-isomerization (Kwon and Armbrust 2005b)

**Fig. 14:**
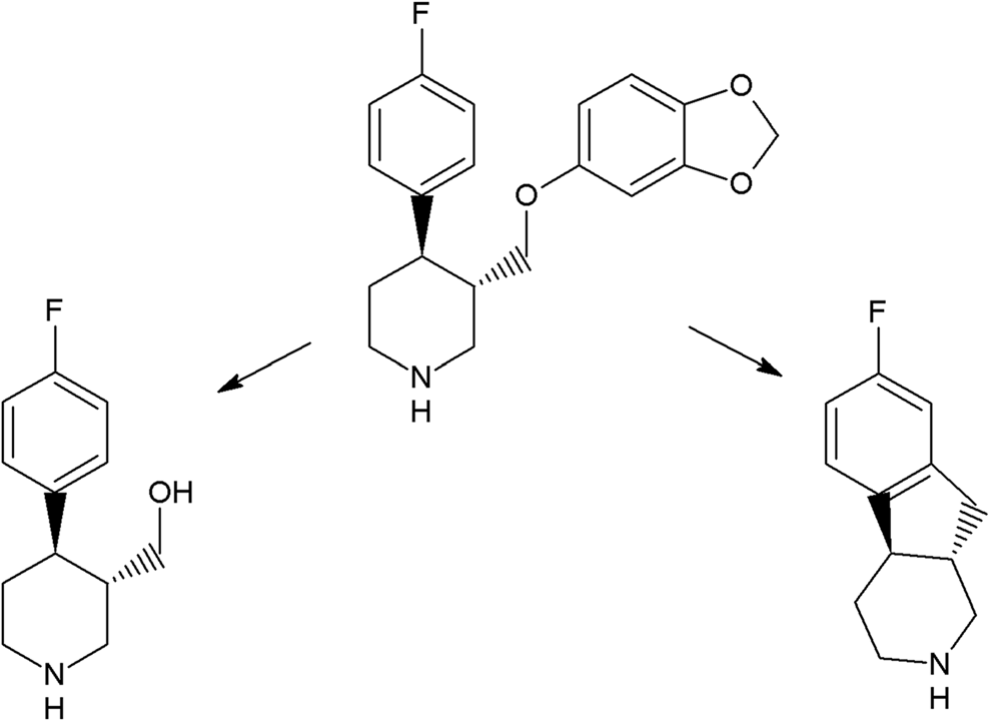
Paroxetine and its photoproducts (Kwon and Armbrust 2004)

Another SSRI antidepressant—citalopram—in acidic and neutral solutions resisted irradiation with simulated solar ultraviolet radiation, but was less stable when dissolved in basic solution. Addition of humic acid or displacement of ultrapure water with river water resulted in increase of the photodegradation rate. Main photoreactions involved N-desmethylation and N-oxidation (Fig. 12) (Kwon and Armbrust 2005a). Similarly, fluvoxamine is more susceptible to indirect photodegradation—isomerization to (Z)-fluvoxamine is six to seven times faster in the presence of DOM than in buffered water (Fig. 13) (Kwon and Armbrust 2005b). The case of paroxetine is substantially different. Comparison of degradation rates in buffered water, lake waters, and synthetic humic water showed that the fastest decomposition took place in basic aqueous solution, while presence of DOM slowed it down (Kwon and Armbrust 2004). Two photoproducts of paroxetine were identified (Fig. 14).

**Fig. 15:**
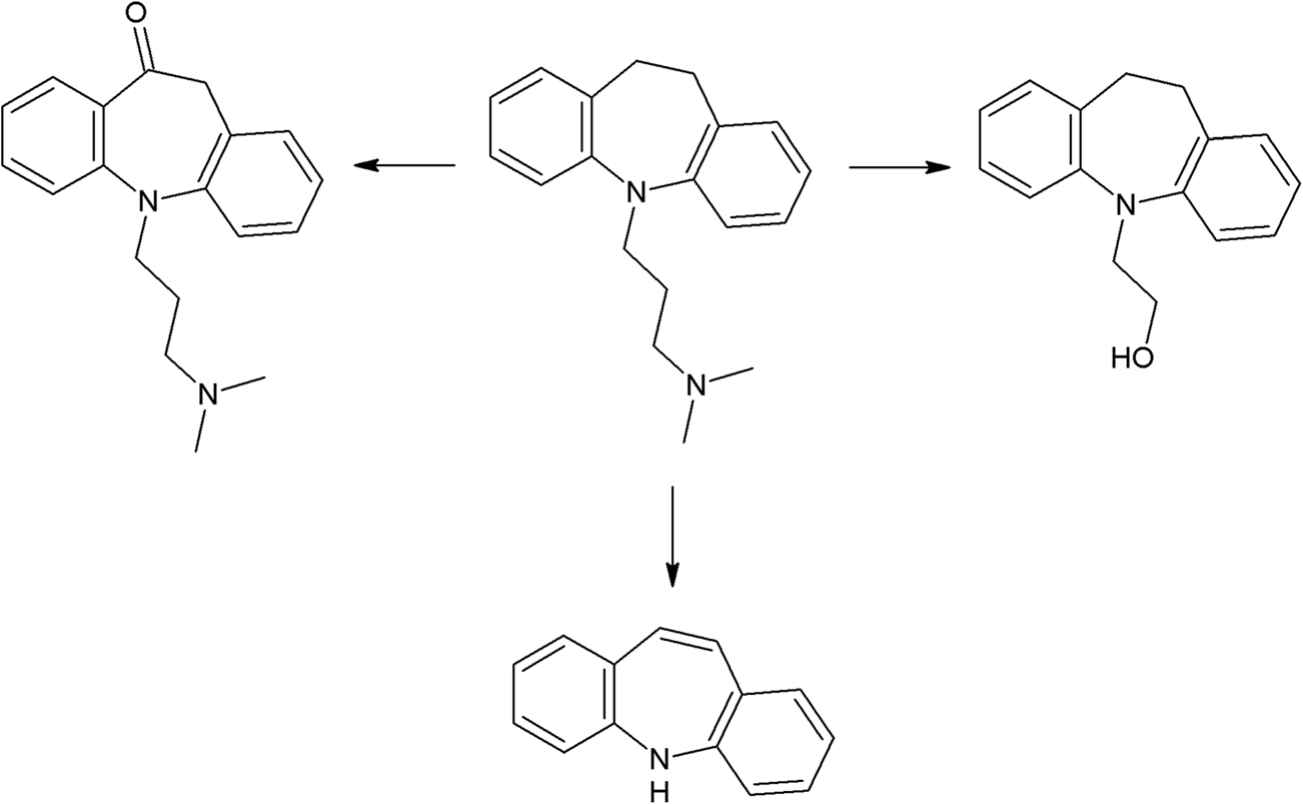
Imipramine and its selected photoproducts (Calza et al. 2008)

**Fig. 16:**
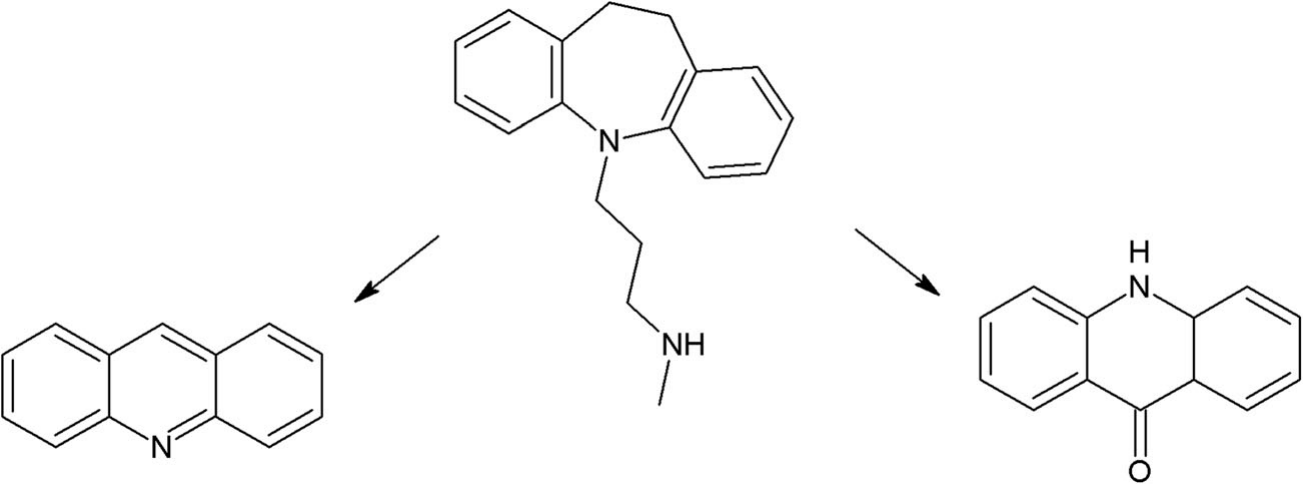
Dezipramine and its photoproducts—acridine and acridine-9(10*H*)-one (Gros et al. 2015)

The first tricyclic antidepressant—imipramine—undergoes rapid photodecomposition under simulated solar irradiation with addition of photocatalysts: H_2_O_2_, Fe(II) compounds, or TiO_2_ which turned out to be most efficient. After 24 h of irradiation, complete mineralization was achieved (75 % after 4 h). Irradiation results in formation of nine identified photoproducts, as an effect of the following reactions: oxidation of a dibenzo[b,f]azepin fragment, hydroxylation, and dehydrogenation or cleavage of a side chain. Selected photodegradants are presented in Fig. 15. Ecotoxicity of irradiated solutions was assessed with use of Microtox bioassay. The increase of the inhibition of bacteria luminescence was observed up to 15 min of irradiation (66 % inhibition at the beginning, 83 % after 15 min). This effect was attributed to the presence of transformation products; however, indicating which one exactly was responsible for toxicity increase was impossible. After reaching its peak, inhibition started to decrease (74 % after 30 min and less than 3 % after 720 min of irradiation) (Calza et al. 2008). Irradiation of desipramine with simulated sunlight in ultrapure water and wastewater resulted in formation of similar products with exception of acridine (Fig. 16) which was not found in the study on imipramine. A considerably higher degradation rate whether wastewater is used as a solvent indicates the major role of indirect photodegradation (Gros et al. 2015).

**Fig. 17:**
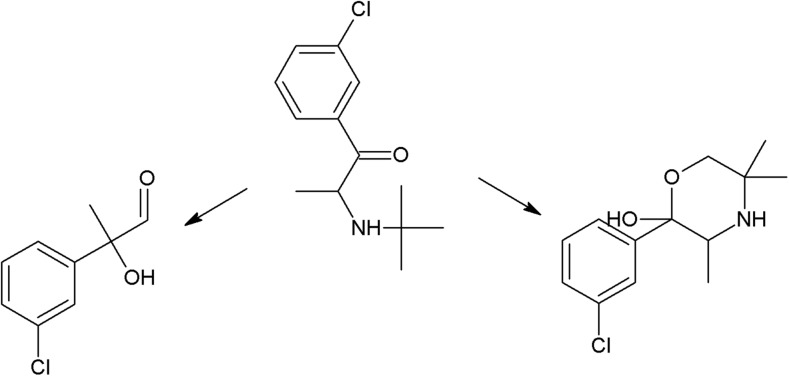
Bupropion and its products after irradiation in basic solution (Bansal et al. 2013)

Bupropion under degradation conditions recommended by ICH is stable in solid state and neutral/acidic solution. However, simultaneous application of radiation and a basic environment lead to formation of five photoproducts. Structures of two of them were elucidated by Bansal et al. (2013) (Fig. 17).

A monoamine oxidase (MAO)-inhibiting agent—moclobemide—is susceptible to UV-C irradiation which leads to formation of 4-chlorobenzamide as a main degradant and two other photoproducts as a consequence of morpholine ring cleavage (Skibiński and Komsta 2012).

**Fig. 18:**
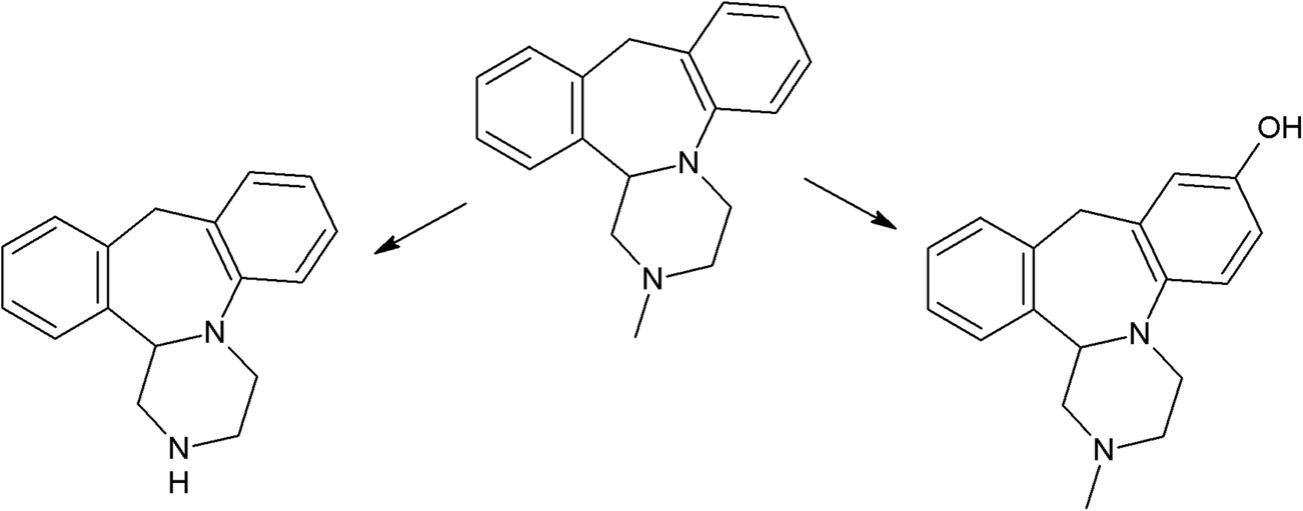
Mianserin and the main products of its photodegradation (Wawryniuk et al. 2015)

Photostability of mianserin under VIS and UV–VIS irradiation has been studied by Wawryniuk et al. (2015). Rapid photodegradation was observed under UV–VIS radiation, while application of VIS radiation did not affect drug molecules. However, after addition of humic acid to the examined solution, mianserin readily decomposed even under VIS irradiation. This effect was attributed to the generation of AOPs by irradiated humic acid. Photodecomposition resulted in formation of three detected products. Structures of two of them are shown in Fig. 18. Toxicity of irradiated samples was assessed by using three tests: Microtox, Spirotox, and Thamnotoxkit F. Photoproducts can be considered as less toxic than the parent molecule. In the other study, irradiation of mianserin with UV-C radiation led to formation of three photoproducts. One of them was identified as *N*-desmethylmianserin. Structures of two remaining products were not identified (Sfair et al. 2012a). Cytotoxic assay of mianserin irradiated with UV-C was done by Sfair et al. by measurement of lactate dehydrogenase releasing into the medium by human mononuclear cells. UV-C-degraded mianserin samples were found as potentially cytotoxic (Sfair et al. 2012b).

**Fig. 19:**
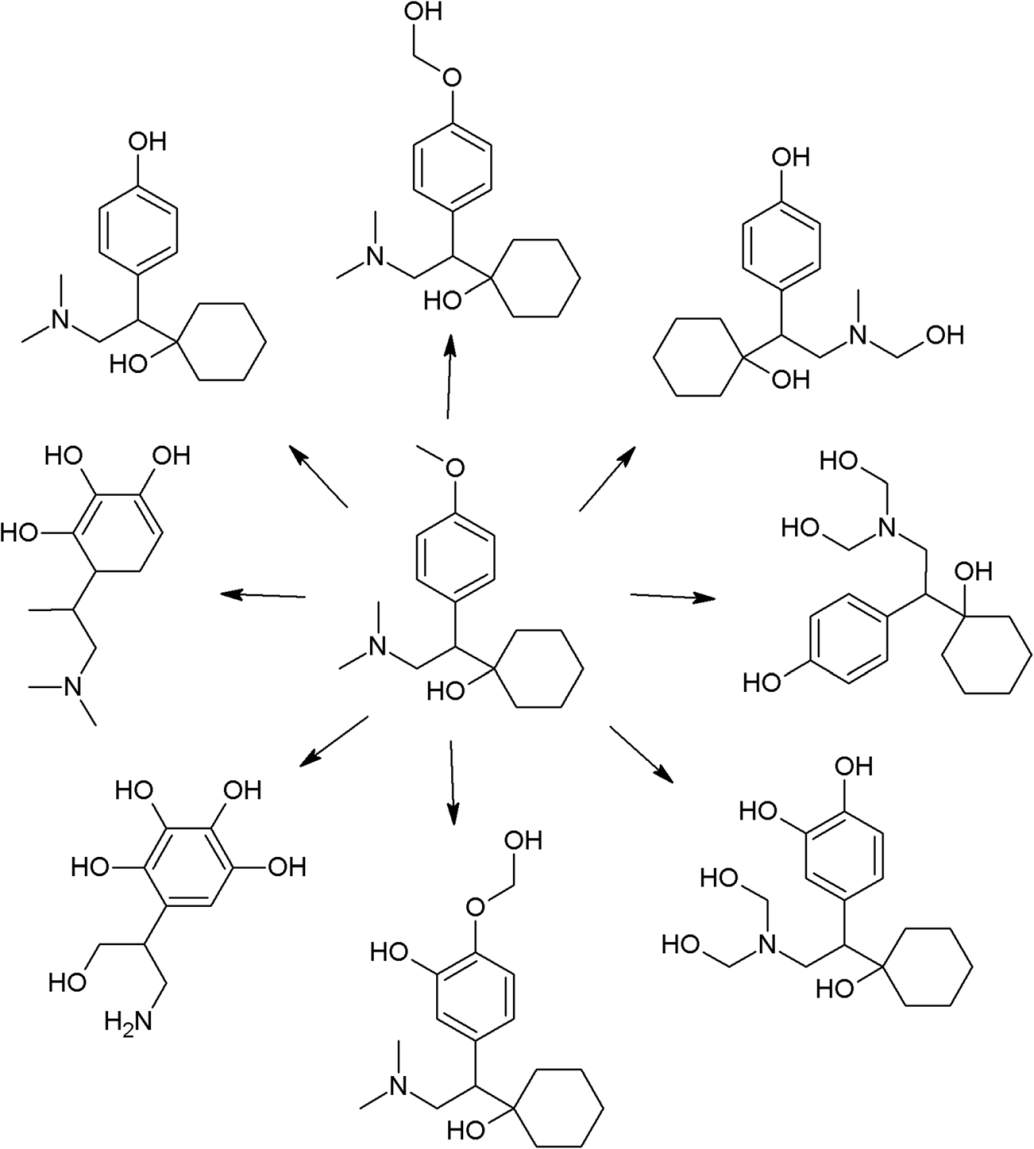
Selected products of phototransformation of venlafaxine (García-Galán et al. 2016)

A study on photodegradation of venlafaxine under a medium-pressure mercury lamp and natural sunlight proved that indirect photodegradation is the most substantial degradation process of this drug, as well as its metabolite *O*-desmethylvenlafaxine. Degradation rates for these two substances in river water when compared to ultrapure water were 8 and 13 times higher respectively. What is important, natural sunlight turned out to be less effective than the mercury lamp (Rúa-Gómez and Püttmann 2013). More than 70 products were detected after UV-A irradiation of venlafaxine with the use of TiO_2_ as photocatalyst. Structures of the most abundant products were elucidated. The drug was completely removed after 240 min of treatment. Microtox bioassay revealed that toxicity of samples was increasing at the beginning of treatment (formation of toxic intermediates) and then started to decrease until the experiment was terminated (Lambropoulou et al. 2016). García-Galán et al. studied degradation of venlafaxine and *O*-desmethylvenlafaxine under UV/H_2_O_2_ (low-pressure mercury lamp) treatment. The applied method allowed rapid elimination of both substances (99.9 % removal after 5 and 30 min respectively). Eleven transformation products were detected and identified (selected are shown in Fig. 19). Toxicity of samples increased in parallel with concentration of photodegradants (Microtox) (García-Galán et al. 2016).

The literature data concerning the photodegradation studies on antidepressants are assembled in Table 3.

**Table 3:** Photodegradation studies on antidepressants

Compound	Medium	Irradiation source	Analysis method	Toxicity assessment	Photoproducts/results	Ref.
Fluoxetine	SS; MetOH; Water; HCl; NaOH	N/A	HPLC	None	2 detected	(Pathak and Rajput 2009)
	Solid state	UV-C	HPTLC	None	None (photostable)	(Shah et al. 2008)
	Water; SFW	290–800 nm	Spectroph. HPLC IC HPLC–MS/MS	None	4 identified	(Lam et al. 2005)
	SS	UV-A	HPLC–MS	None	2 identified	(Maalanka et al. 2013)
	Water + Ga_2_O_3_	UV-C	IC UV–VIS	Ames Test	2 photoproducts (defluorination and oxidation)	(Hidaka et al. 2013)
	Water + TiO_2_	UV–VIS	Spectroph.	None	None	(da Silva et al. 2015)
	Water; O_3_; TiO_2_; H_2_O_2_	Hg lamp (360 nm)	HPLC–MS	None	4 identified	(Méndez-Arriaga et al. 2011)
Fluvoxamine	Water + Ga_2_O_3_	UV-C	IC UV–VIS	None	1 (product of defluorination)	(Hidaka et al. 2013)
	Aqueous buffers; SHW	UV sunlight simulation	Spectroph. UV–VIS HPLC–MS	None	1 identified	(Kwon and Armbrust 2005a)
	SS; Water	UV VIS	HPLC	None	None (unstable especially in solution under UV irradiation)	(Souri et al. 2015)
Citalopram	Aqueous buffers; Lake water	UV sunlight simulation	Spectroph. UV–VIS HPLC–MS	None	2 identified	(Kwon and Armbrust 2005b)
	SS; Water; HCl; NaOH	ICH	HPLC–PDA–MS/MS IR	None	3 (one identified—N-oxide)	(Sharma et al. 2011)
	Water	UV	Spectroph.	None	None (photostable)	(Tapkir et al. 2016)
Escitalopram	ACN-buffer	ICH	HPLC–PDA	None	None (photostable)	(Kakde et al. 2013)
Paroxetine	Water; River waters; H_2_O_2_	Hg lamps	UHPLC–MS/MS	None	None	(Tixier et al. 2003)
	Aqueous buffers; SHW; Lake waters	UV sunlight simulation	Spectroph. HPLC–MS	None	2 identified	(Kwon and Armbrust 2004)
	MetOH	Natural sunlight	HPTLC	None	None (photostable)	(Venkatachalam and Chatterjee 2007)
Sertraline	MetOH	Direct sunlight	HPTLC	None	1 detected	(Hussain et al. 2013)
	MetOH	245 nm	Spectroph. Spectrofluorimetric	None	None (photostable)	(Walash et al. 2011a, b)
Bupropion	Water–MetOH; HCl; NaOH	N/A	HPLC–PDA HPLC–MS	None	5 (2 identified)	(Bansal et al. 2013)
Moclobemide	MetOH	UV-C	Spectroph. HPLC–MS	None	3 identified	(Skibiński and Komsta 2012)
	MetOH	Natural sunlight	HPTLC	None	None (photostable)	(Patel et al. 2008)
Dosulepin	SS	N/A	HPLC	None	None	(Reddy et al. 2014)
Tianeptine	SS	UV-C	TLC HPLC GC–MS	None	4 detected	(Khedr 2007)
Duloxetine	MetOH; SS	Natural sunlight; UV	HPLC	None	4 detected	(Patel et al. 2010)
	ACN	UV-C	HPLC	None	4 detected	(Gomes et al. 2010)
	MetOH	Natural sunlight	HPTLC	None	1 detected	(Patel et al. 2009)
	Buffer–ACN–MetOH	254 nm	HPLC–PDA	None	None (mild photodegradation)	(Srinivasulu et al. 2009)
	Water–MetOH	ICH	HPLC–MS	None	None (photostable)	(Kumar et al. 2012)
	MetOH	Natural sunlight	HPLC	None	None (photostable)	(Chhalotiya et al. 2010)
	MetOH–water	ICH	UPLC–PDA	None	None (photostable)	(Rao et al. 2010)
	Buffer–ACN	ICH	HPLC–PDA	None	None (photostable)	(Raman et al. 2010)
	Buffer–ACN	ICH	HPLC–PDA	None	None (photostable)	(Veera et al. 2012)
Venlafaxine	Water; River water	Hg lamp (290–600 nm); Natural sunlight	Specroph. GC–MS	None	None	(Rúa-Gómez and Püttmann 2013)
	Buffer–ACN	UV	HPLC–PDA	None	None (mild photodegradation)	(Rao et al. 2015)
	Water (various pH) + TiO_2_	UV-A	UPLC–MS–LIT–Orbitrap IC TOC analyzer	Microtox (*V. fisheri*)	Over 70 detected (4 identified)	(Lambropoulou et al. 2016)
	Water + H_2_O_2_	254 nm	UPLC–MS–LTQ–Orbitrap	Microtox (*V. fisheri*)	11 identified	(García-Galán et al. 2016)
Imipramine	Water	UV-A	Spectroph. HPLC	RBC lysis; 3 T3 Test; PBR322 DNA strand breaks	None	(Viola et al. 2000)
	Water + H_2_O_2_, Fe (II), TiO_2_	300–800 nm	HPLC–MS GC–MS	Microtox	9 identified	(Calza et al. 2008)
Desipramine	Water; WW	300–800 nm	UHPLC–MS	Microtox	10 identified	(Gros et al. 2015)
	Water–ACN	ICH	HPLC	None	None (photostable)	(Thiyagarajan et al. 2008)
	Water (various pH)	Xenon; UV	UPLC–MS–LTQ–Orbitrap	CBT, MRT tests Computational toxicity prediction	18 identified	(Khaleel et al. 2016)
Mianserin	Water; SHW	300–800 nm	HPLC–MS/MS	Microtox Spirotox Thamnotoxkit	3 (2 identified)	(Wawryniuk et al. 2015)
	MetOH	UV-C	MEKC MS	None	2 (1 identified)	(Sfair et al. 2012a)
	MetOH	UV-C	HPLC	LDH activity test	None	(Sfair et al. 2012b)
Maprotiline	Buffer–ACN–MetOH–THF	ICH	HPLC–DAD	None	Several detected	(Đurić et al. 2015)
Agomelatine	MetOH	254 nm	HPLC	None	None (photostable)	(El-Shaheny 2014)
Vilazodone	Water	ICH	UPLC–MS/MS	None	None (photostable)	(Kalariya et al. 2015)

**Fig. 20:**
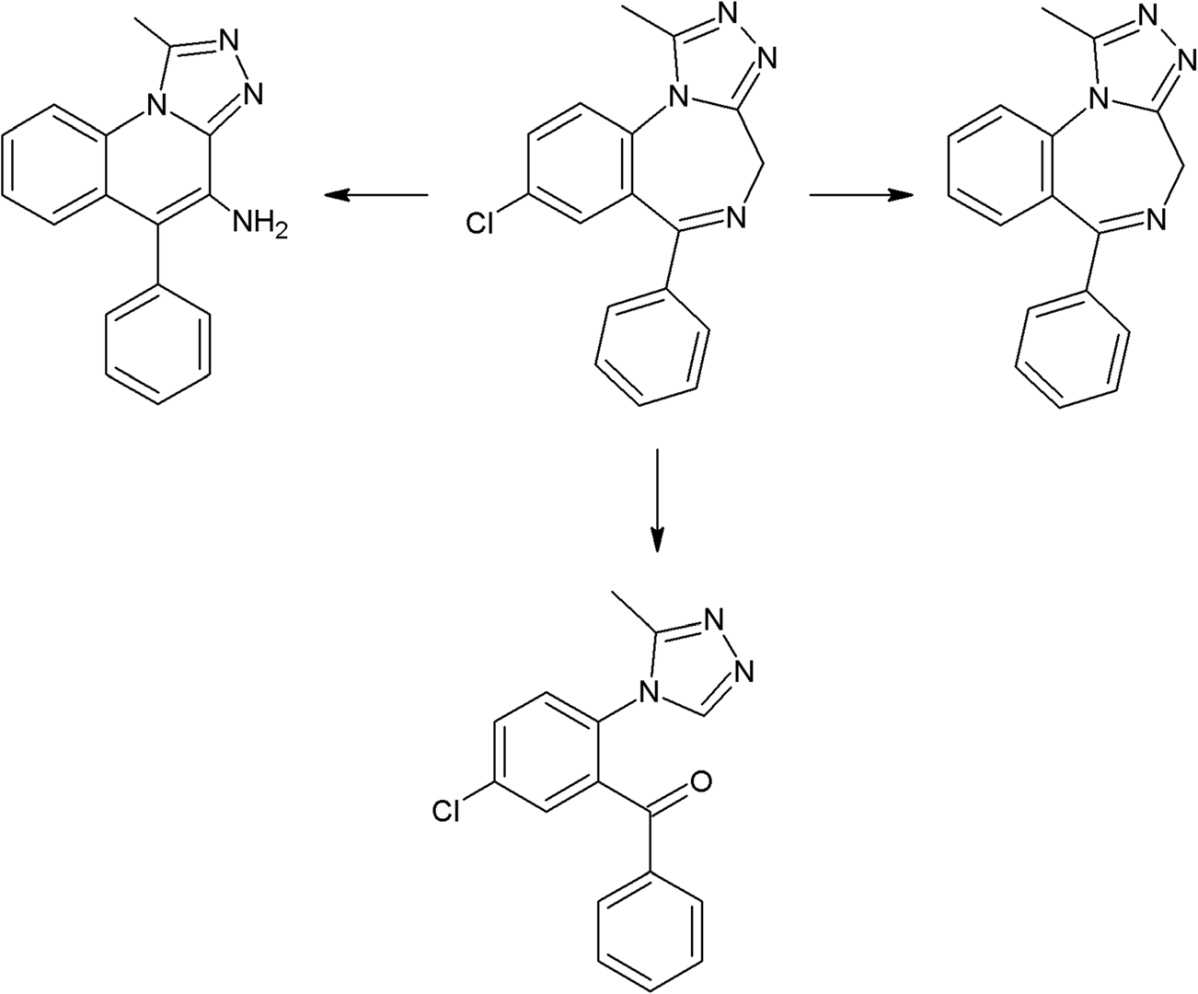
Alprazolam and its photoproducts (Nudelman and Carbera 2002)

**Fig. 21:**
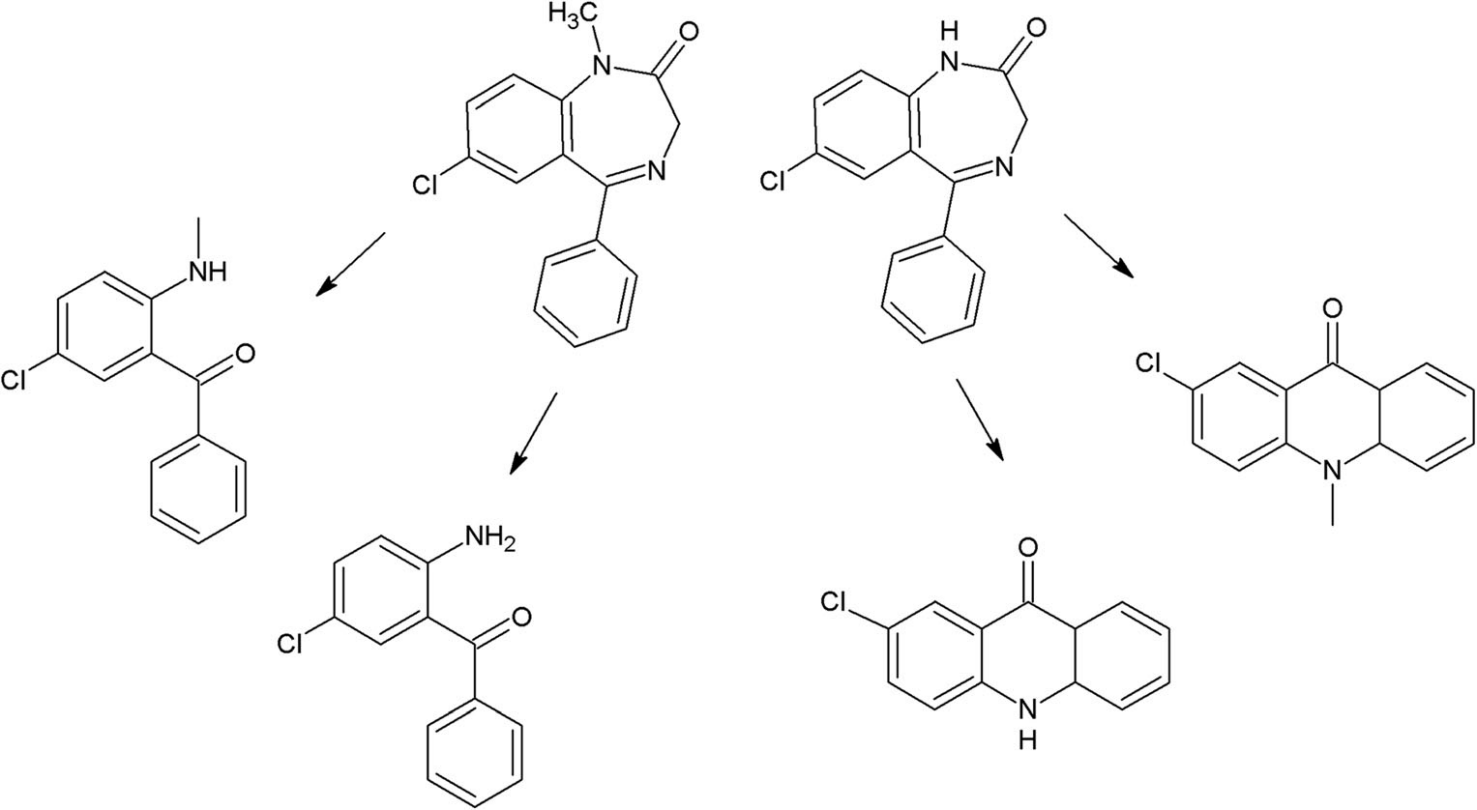
Diazepam, nordiazepam, and their photoproducts (West and Rowland 2012)

Taking into account anxiolytic drugs, benzodiazepines have the most studied photochemistry. Pathak and Rajput (2008) reported high susceptibility of alprazolam to photolytic conditions, especially in acidic medium. After 15 days of irradiation in a photostability chamber, 30–35 % degradation was observed with formation of five main photoproducts (their structures were not elucidated). According to research performed by Nudelman and Cabrera, the rate of photolysis of alprazolam is pH dependent. In basic solutions (pH = 9), photodegradation did not occur at all, while in acidic solutions, alprazolam was photolabile. Moreover, the rate of photodegradation was about two times faster in pH 2 than pH 3.6. Three main photoproducts were identified in this case (Fig. 20) (Nudelman and Cabrera 2012). The main identified photoproduct—triazoloquinoeine—is a highly fluorescent compound, whose formation may be responsible for the photosensivity observed in patients treated with alprazolam (Cabrera et al. 2005). On the contrary, a study by Calisto et al. proved that alprazolam is highly photostable with *t*_1/2_ over 800 h in water. An indirect photodegradation experiment was terminated after 48 h of irradiation, and lack of observable decomposition has been seen although two photoproducts were identified—3-chloro-(3-methyl-3*H*-1,2,4-triazol-4-yl)benzophenone and hydroxylated 7-chloro-5-phenyl-3,3a,4,5-tetrahydro-1,2,4-triazol[4,3-a]quinoline (Calisto et al. 2011b). The difference between this finding and those by Cabrera et al., as well as Nudelman and Cabrera, may be the consequence of different pH of alprazolam solutions. In light of the fact that photo-resistance of alprazolam increases in parallel with solution basicity, such explanation could be possible (nevertheless, an explanation of results of a photolysis experiment by Pathak and Rajput is more problematic). According to the same study, diazepam is a more photolabile molecule, with *t*_1/2_ = 28 h in pure water. To assess indirect photodegradation, three different factions of humic substances (humic acids, fulvic acids, and XAD4) were added to the solutions. Fulvic acids and XAD4 caused an increase of the photodegradation rate, while addition of humic acids was responsible for inhibition of photodecomposition of diazepam, as well as other studied benzodiazepines. Four photoproducts were identified—two indole derivatives, one derivative of isoquinoline, and 2-chloro-5-methyl-10,11-dihydro-5*H*-dibenzo[b,f]azepine (Cabrera et al. 2005). West and Rowland also studied direct and indirect photodegradation of diazepam as well as its metabolites—nordiazepam, temazepam, and oxazepam (West and Rowland 2012). Half-lives were estimated 100 and 200 h for diazepam and nordiazepam respectively, while half-lives of oxazepam and temazepam were considerably lower (36 and 32 h respectively), which is probably a consequence of few times higher quantum yields. Addition of humic substances increased photodecomposition rates of diazepam and nordiazepam (28 and 47 h respectively). An interesting fact is that the humic substances slow down the process of photodegradation of temazepam and oxazepam, probably as an effect of screening. The total number of photodegradants was 14; four of them were products of irradiation of diazepam (Fig. 21). None of them were the same as identified by Calisto et al. (2011b).

**Fig. 22:**
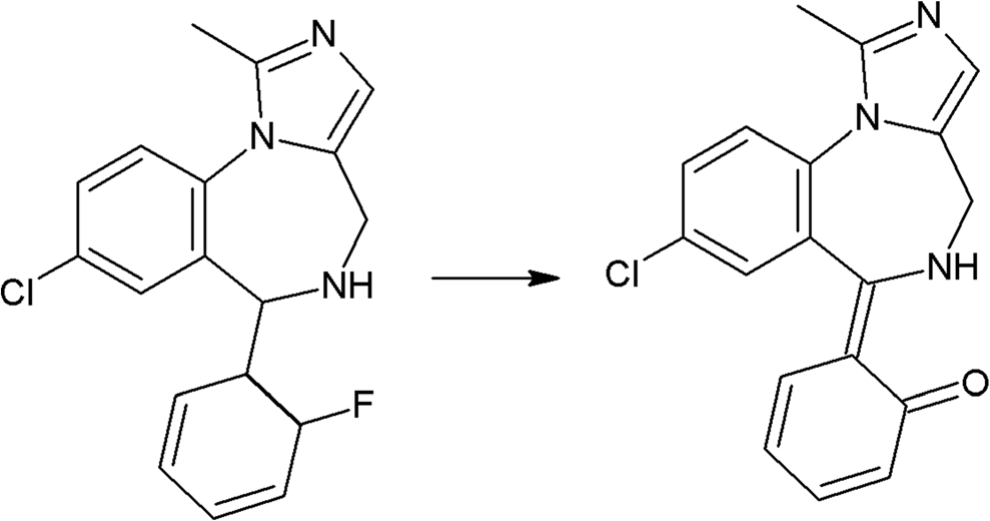
Midazolam and the product of its photodegradation with the use of a high-pressure mercury lamp (Andersin et al. 1994)

Sousa et al. proposed a three-step pathway of photodecomposition of lorazepam (with or without addition of TiO_2_), using two lab-scale photoreactors (one equipped with a medium-pressure mercury lamp, the second equipped with a blacklight blue lamp), and under natural solar irradiation. The method using natural sunlight with titanium dioxide (200 mg L^−1^) turned out to be most efficient. Six main photoproducts were detected (in the first step), mainly as a consequence of cleavage of a diazepinone ring followed by a rearrangement into a highly stable quinazoline structure, and then its modifications (e.g., hydroxylation). Another transformation route involved hydroxylation of the benzodiazepine ring system. The second step consists of hydroxylation of 6-chloro-4-(2-chlorophenyl)-3*H*-quinazoline in various positions and substitution of chlorine by a hydroxyl group. The third step involved further transformation of substituted quinazoline, e.g., formation of fluorene or benzophenone derivatives (Sousa et al. 2013b). Concurrently, Calisto et al. report lorazepam as the most photolabile compound amongst all studied benzodiazepines with *t*_1/2_ = 2.6 h (direct photodegradation). Six main photoproducts were identified, but only two of them were identical to those detected by Sousa et al. The four different were chlorinated derivatives of acridine, dibenzo[b,f]-1,4-diazepine, and benzisoxazole. In the case of oxazepam, seven photoproducts were detected and identified: derivatives of quinoline, quinazoline, benzophenone, and the parent structure with a hydroxylated phenyl functional group. One of the photodegradants, different to those typical of other benzodiazepines, was 2-isocyanatebenzonitrile. The half-life of oxazepam was estimated as six times longer than that of lorazepam, despite only slight difference in structure between these two molecules (Calisto et al. 2011b). The type of formed photoproducts of midazolam depends on whether a mercury lamp or natural solar irradiation was used. Under a high-pressure mercury lamp, the parent molecule undergoes defluorination, followed by oxidation and formation of ketone as a result (Fig. 22). A quinazoline derivative is formed as a consequence of irradiation with the mercury lamp, as well as an action of daylight (Andersin et al. 1994). Photostability of midazolam is pH dependent—the lowest photodecomposition rate was observed at pH 1.3, while at pH 6.4, the molecule was least stable. This phenomenon is probably connected with the formation of a molecule-stabilizing open-ring form of midazolam which outweighs a closed-ring form in highly acidic medium (Andersin and Tammilehto 1995).

**Fig. 23:**
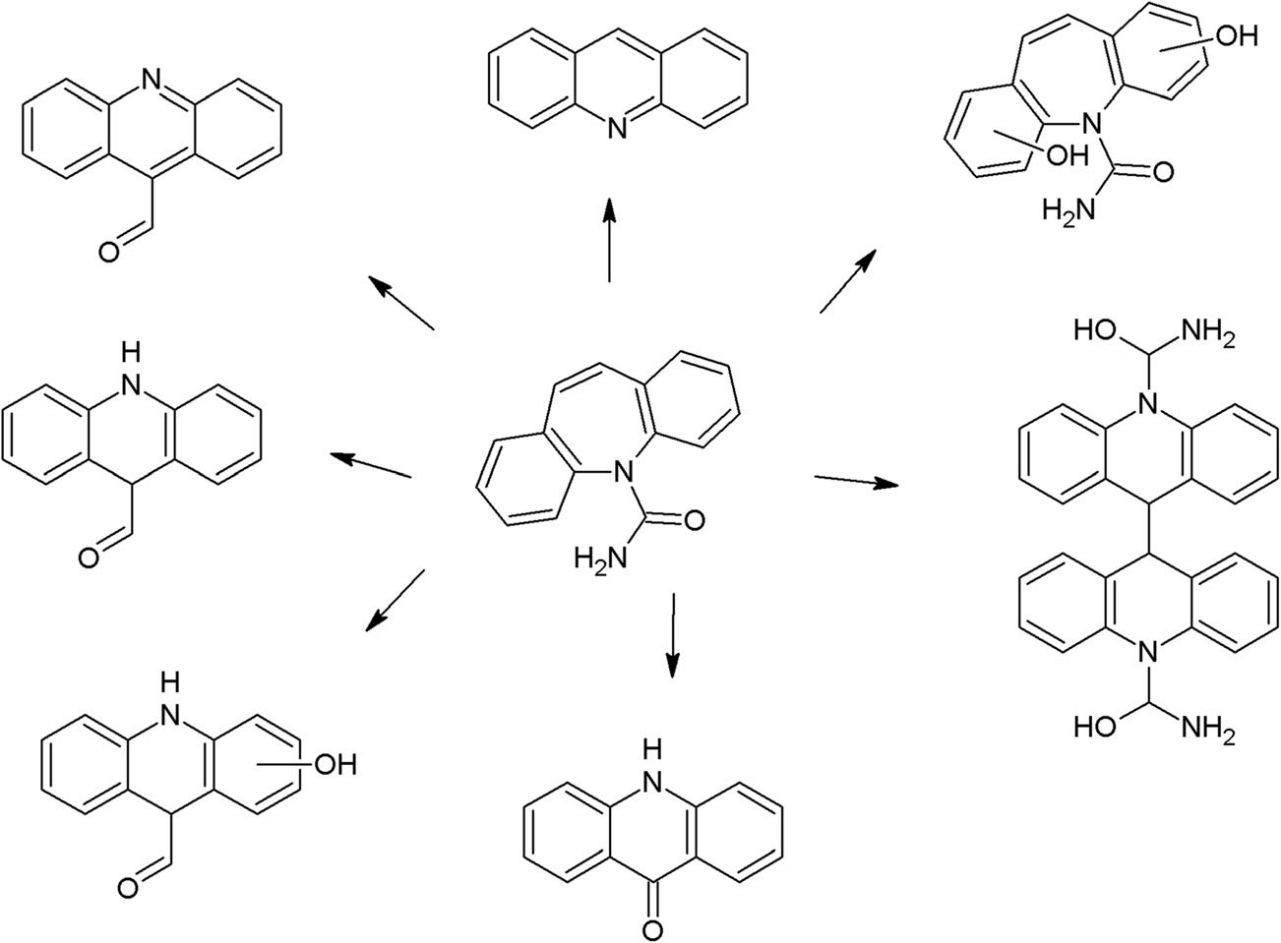
Carbamazepine and products of its direct photolysis (Calisto et al. 2011a)

**Fig. 24:**
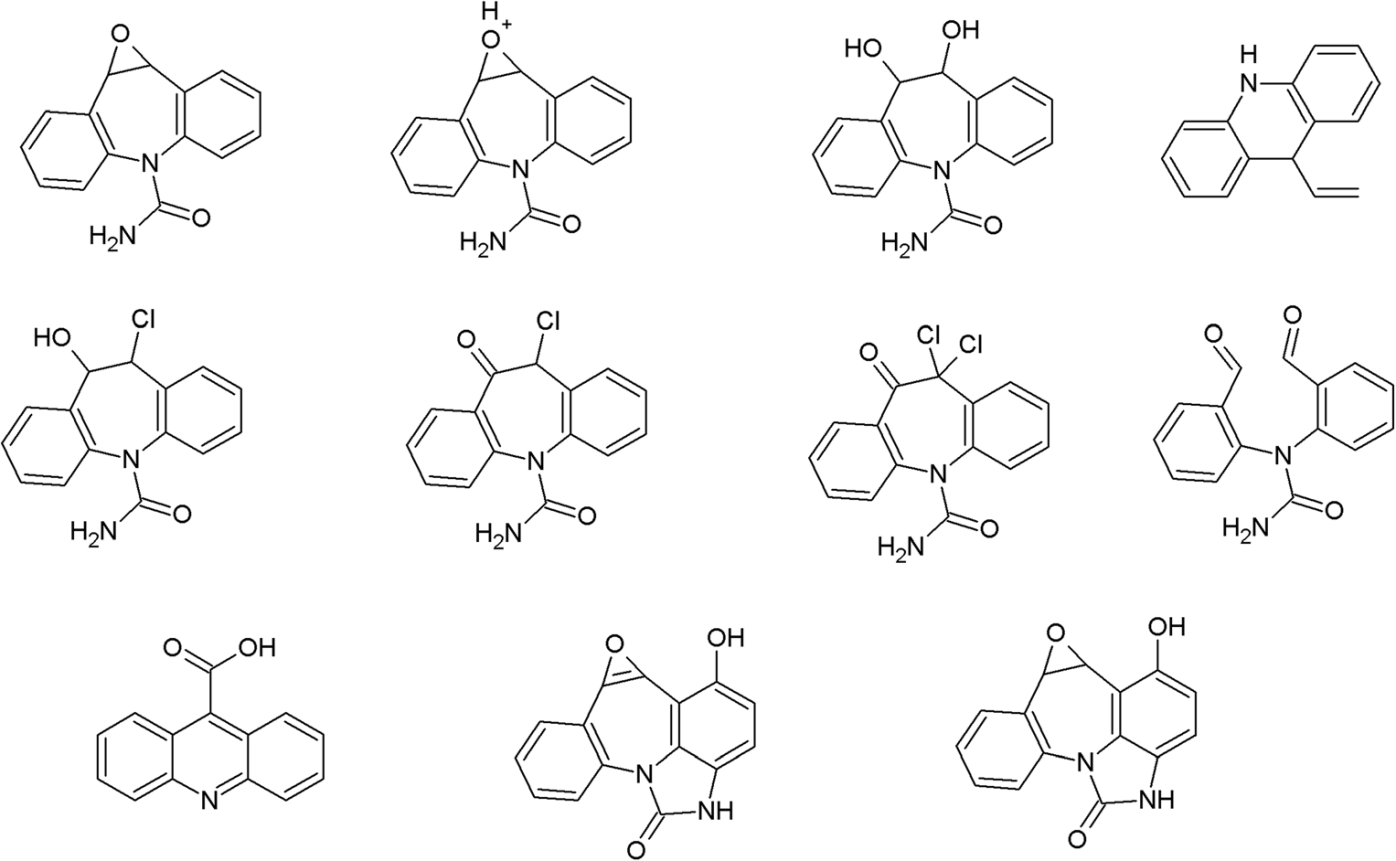
Products of UV/Cl_2_ treatment of carbamazepine (Wang et al. 2016; Zhou et al. 2016)

**Fig. 25:**
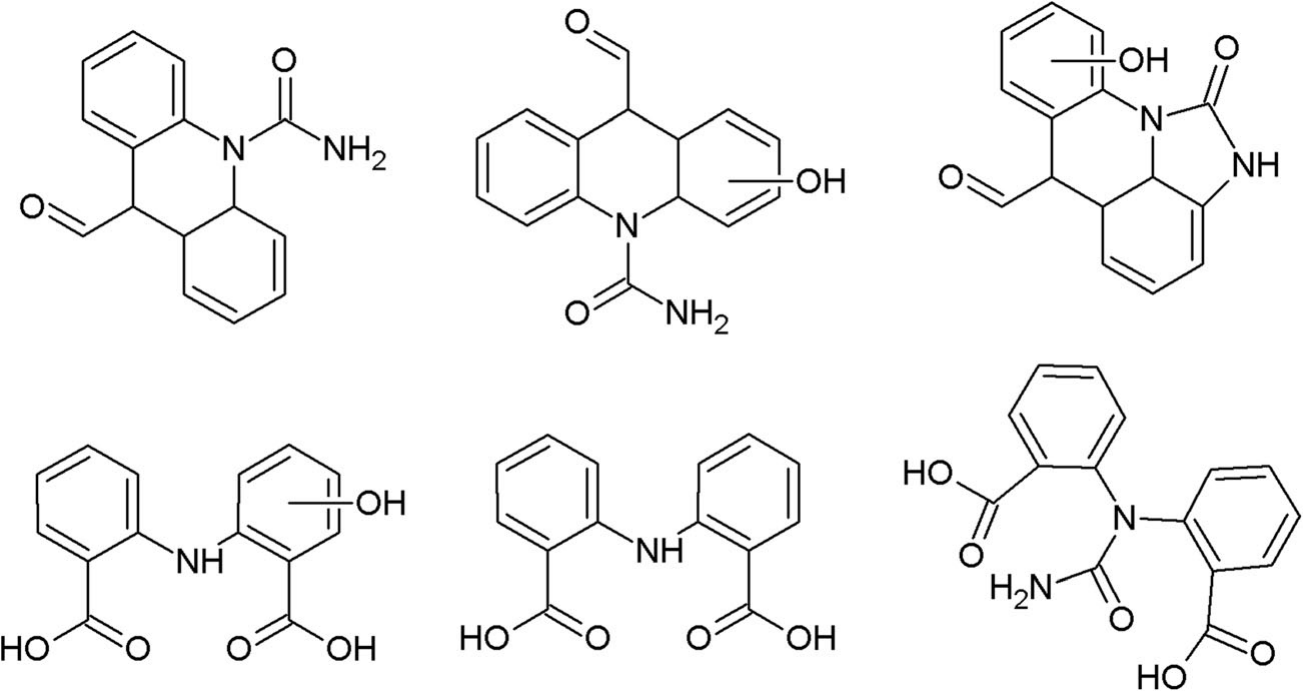
Phototransformation products of carbamazepine after treatment with the use of bismuth-based photocatalysts (Xu et al. 2013; Gao et al. 2015)

Taking into account high consumption and common occurrence in the environment, photodegradation of carbamazepine was a subject of numerous studies. Calisto et al. investigated direct photolysis of carbamazepine dissolved in ultra pure water (at different pH and oxygenation levels) under simulated solar radiation. The compound was most photolabile at the lowest pH (2.9) and low oxygenation level. At pH = 9, the drug was most stable; moreover, its degradation was no longer dependent on oxygenation of solution. Seven photoproducts were detected, and their structures were elucidated (Fig. 23). Most of them were acridine derivatives (Calisto et al. 2011a). Several different photoproducts were formed in UV/Cl_2_ photolysis processes, including carbamazepine epoxide, chlorinated products, or four-membered ring derivatives (Fig. 24). Acridine derivatives were found as well (Wang et al. 2016; Zhou et al. 2016). Lekkerkerker-Teunissen et al. reported resistance of carbamazepine both to UV (low- and medium-pressure mercury lamps) and UV/H_2_O_2_ treatment in deionized water. Nevertheless, six phototransformation products were detected (carbamazepine epoxide and acridine derivatives) (Lekkerkerker-Teunissen et al. 2012). On the contrary, according to a study by Liu et al., carbamazepine was effectively removed by UV and UV/H_2_O_2_ photolysis also in distilled water (low-pressure mercury lamp was applied). Combination of UV radiation and Fe(II) ions, which was also studied, gave the best results. Seven photoproducts were detected and identified: hydroxylated carbamazepine derivatives, carbamazepine epoxide, and acridine derivatives (Liu et al. 2014). Different findings of these two studies are difficult to explain, taking into consideration similar applied conditions of photolysis. Additionally, numerous studies on photocatalytic treatment of carbamazepine with the use TiO_2_ were published. The use of bare P90 TiO_2_ and UV-A radiation resulted in 94.67 % of carbamazepine removal after 90 min of experiment (distilled water). In wastewater effluent, the degradation rate reached 96.57 %. Nine photoproducts were identified (previously reported epoxide, hydroxylated derivatives, and several acridine derivatives) (Carabin et al. 2016). Carabin et al. compared effectiveness of carbamazepine removal with the use of five TiO_2_ photocatalysts (P90, P25, UV100, PC500, and ST01) under 365 nm irradiation. Experiments were conducted in aqueous solutions, and P90 was the most effective (Carabin et al. 2015). Application of TiO_2_-coated nanosilica-cobalt ferrite (CoFe_2_O_4_/SiO_2_/TiO_2_) composites resulted in a higher photodegradation rate than in the case of P25 TiO_2_. Carbamazepine was totally removed from the tap water sample after 60 min of irradiation (254 nm); however, residues were detected in the distilled water sample (Nazarkovsky et al. 2016). Ten photoproducts (epoxide, hydroxylated, four-membered-ring, and acridine derivatives) were formed in the photodegradation experiment with the use of P25, ZnO, and multi-walled carbon nanotubes–anatase composites. Application of P25 gave the best results (Martínez et al. 2011). Three previously reported products (epoxide, hydroxy- and dihydroxycarbamazepine) were detected after photocatalytic treatment of carbamazepine with the use of whey-stabilized TiO_2_ and ZnO under UV-C irradiation. Higher photodegradation (100 % after 55 min) was observed by using TiO_2_ (ZnO 92 %). Estrogenic activity of photocatalyzed samples was assessed with the use of the yeast estrogen test (YES). Results of the test were negative (non-estrogenic) (Mohapatra et al. 2014). The toxicity of carbamazepine and its photoproducts (seven detected, all previously reported) after sonolytic and sonophotocatalytic treatment (in pure, ground, and wastewater) was investigated by Jelic et al. Both photocatalyzed and non-photocatalyzed samples were highly toxic to *D. magna* (Jelic et al. 2013). Anthranilic acid and acridine were detected as products of photoelectric treatment with the use of Ti/TiO_2_ electrodes. Microtox bioassay proved that photocatalyzed samples were not toxic (Daghrir et al. 2013). Several previously unreported phototransformation products (Fig. 25) were identified in a study on degradation of carbamazepine with bismuth-based photocatalysts (BiOCl and BiPO_4_). In the case of BiPO_4_ the drug was completely removed from ultra pure water after 60 min of treatment (mercury lamp was used) (Xu et al. 2013). (Gao et al. 2015). Comparison of toxicity of carbamazepine solution before, during, and after direct photolysis treatment revealed that phototransformation products were more toxic than the parent compound. Toxicity of samples to *V. fisheri*, green algae *Pseudokirchneriella subcapitata*, and *D. magna* was increasing in parallel with concentration of acridine and acridone (Donner et al. 2013).

The literature data concerning the photodegradation studies on anxiolytics and carbamazepine are assembled in Table 4.

**Table 4:** Photodegradation studies on anxiolytics and carbamazepine

Compound	Medium	Irradiation source	Analysis method	Toxicity assessment	Photoproducts/results	Ref.
Alprazolam	Water–MetOH; SS	Black light UV; Fluorescent lamps	HPLC	None	5	(Pathak and Rajput 2008)
	Buffered water–MetOH	Metal halide lamp (350–650 nm)	HPLC ^1^H NMR	None	3 identified	(Nudelman and Cabrera 2002)
	Buffered water–MetOH	Metal halide lamp (350–650 nm)	GC–MS ^1^H NMR	None	3 identified	(Cabrera et al. 2005)
	Acidic buffers	Metal halide lamp (350–650 nm)	MS ^1^H NMR ^13^C NMR	None	3 identified	(Nudelman and Gallardo 2002)
	Water + TiO_2_	Hg lamp	HPLC	None	None	(Tomić et al. 2015)
	Water	N/A	MEKC MS	None	2 identified	(Calisto et al. 2011b)
Diazepam	Water; Water + DOM	N/A	MEKC MS	None	4 identified	(Calisto et al. 2011b)
	Water; HW	300–800 nm	Spectroph. HPLC–MS^n^	None	4 identified	(West and Rowland 2012)
Temazepam	Water; HW	300–800 nm	Spectroph. HPLC–MS	None	5 identified	(West and Rowland 2012)
Oxazepam	Water; Water + DOM	N/A	MEKC MS	None	7 identified	(Calisto et al. 2011b)
	Water; HW	300–800 nm	Spectroph. HPLC–MS	None	5 identified	(West and Rowland 2012)
Lorazepam	Water; Water + DOM	N/A	MEKC MS	None	6 identified	(Calisto et al. 2011b)
	Water; WW + TiO_2_	UV-C; Natural sunlight	UHPLC–MS	None	6 identified	(Sousa et al. 2013b)
	Water + TiO_2_	UV-C; Natural sunlight	HPLC – MS/MS	None	None	(Sousa et al. 2013a)
	WW + TiO_2_	Natural sunlight	SPE–HPLC–MS GC–MS	ToxAlert (*V. fischeri*)	None	(Sousa et al. 2012)
Midazolam	Aqueous buffers	Hg lamp; Natural sunlight	Spectroph. HPLC	None	3 identified	(Andersin and Tammilehto 1995)
Clobazam	Water; SS	UV–VIS	HPLC	None	None	(Souri et al. 2014)
Clorazepate	SS	Natural sunlight	HPLC–DAD HPLC–MS		1 identified (*N*-desmethyldiazepam)	(Burana-Osot et al. 2015)
Chlordiazepoxide	Water; HCl; NaOH; SS	N/A	HPLC	None	2 major photoproducts	(Pathak et al. 2010)
Etifoxine	MetOH	UV	HPLC–MS/MS ^1^H NMR ^13^C NMR	None	2 identified	(Djabrouhou and Guermouche 2014)
Meprobamate	Water; WW	Natural sunlight	SPE–HPLC–MS/MS HPLC TOC analyzer	None	None	(Dong et al. 2015)
	SS MetOH	UV; Fluorescent light	LC–MS (IT)	None	None (photostable)	(Karthikeyan et al. 2010)
Hydroxyzine	ACN–MetOH–buffer	Natural sunlight	HPLC	None	None (photostable)	(Sher et al. 2015)
Buspirone	SS Water	Daylight	HPLC	None	Several detected	(Khedr and Sakr 1999)
Carbamazepine	Water (various pH)	Xenon (cutoff below 290 nm)	MEKC MS/MS	None	7 identified	(Calisto et al. 2011a)
	Water (various pH); Water + NOM; Water + Cl_2_	254 nm	HPLC–MS	None	8 identified	(Zhou et al. 2016)
	Water (various pH); WW; Water + Cl_2_	254 nm	SPE–HPLC–MS/MS	None	9 identified	(Wang et al. 2016)
	Water (various pH); WW; US	254 nm	HPLC–MS/MS	*Chlorella vulgaris*	21 identified	(Rao et al. 2016)
	Water	Xenon (cutoff below 290 nm)	HPLC–MS/MS CE	*Scrobicularia plana*; GLYC; PROT; LPO; SOD activity; GSTs activity	5 identified	(Almeida et al. 2016)
	Water	185–400 nm	SPE–GC–MS	Biotox (*V. fisheri*); *D. magna*; *Pseudokirchneriella subcapitata*	Acridine and acridone	(Donner et al. 2013)
	MetOH	Natural sunlight	HPLC	None	1 detected	(Rajadhyaksha et al. 2007)
	Water (various pH); Water + DOM; Water + H_2_O_2_ Water + Fe(II)	254 nm	HPLC–MS/MS IC	None	7 identified	(Liu et al. 2014)
	Water (various pH);	Hg lamps (200–800 nm)	LC–MS/MS	None	6 identified	(Lekkerkerker-Teunissen et al. 2012)
	Water + H_2_O_2_	254 nm; Natural sunlight	HPLC GC–MS	None	6 main identified	(Vogna et al. 2004)
	Water (various pH) + TiO_2_; WW + TiO_2_	UV-A	LC–MS/MS	None	9 identified	(Carabin et al. 2016)
	Distilled water and tap water + CoFe_2_O_4_/SiO_2_/TiO_2_	254 nm	HPLC	None	None	(Nazarkovsky et al. 2016)
	Water (various pH) + 5 TiO_2_ catalysts	365 nm	LC–MS/MS	None	Acridine and other (unidentified)	(Carabin et al. 2015)
	Water and WW + whey stabilized TiO_2_ and ZnO	254 nm	LC–MS/MS	YES Test	3 identified	(Mohapatra et al. 2014)
	Water and WW + TiO_2_, H_2_O_2_ and US	UV-A; Natural sunlight	UPLC–MS/MS	*D. magna*	7 identified	(Jelic et al. 2013)
	Water + TiO_2_	360 nm	LC–LTQ–Orbitrap IC TOC analyzer	None	28 identified	(Calza et al. 2012)
	Water + Ti/TiO_2_ electrocatalysis	254 nm	LC–MS/MS	Microtox (*V. fisheri*)	2 identified	(Daghrir et al. 2013)
	Water + TiO_2_, ZnO, carbon nanotubes–anatase composites, H_2_O_2_	Low and medium pressure Hg lamps	Spectroph. HPLC–LTQ–Orbitrap	None	10 identified	(Martínez et al. 2011)
	Water (various pH) + BiOCl microspheres	Xe lamp	HPLC–MS^n^	None	9 identified	(Gao et al. 2015)
	Water + BiPO_4_	Hg lamp	HPLC–MS/MS	None	10 identified	(Xu et al. 2013)
Oxcarbazepine	ACN–MetOH	254 nm	HPLC–PDA	None	None (photostable)	(Pathare et al. 2007)
	Water; Synthetic surface water	254 nm	SPE–HPLC–MS/MS	*D. magna*	5 identified (photostable in direct photolysis experiment)	(Li et al. 2011)

## Analytical methods used in photodegradation studies

**Fig. 26:**
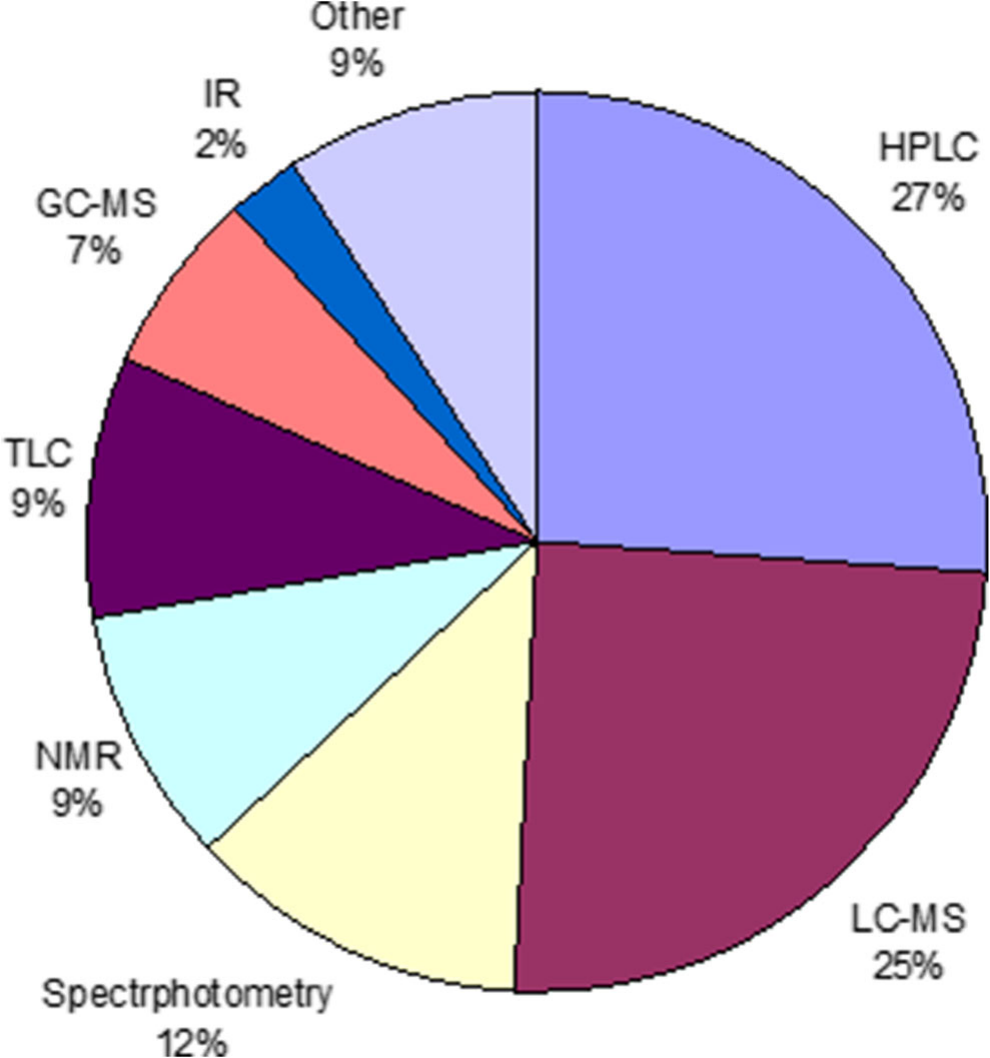
Analytical methods used in photodegradation studies on psychotropic pharmaceuticals

In the case of researches on photodegradation of pharmaceuticals, chromatographic methods, especially HPLC, are methods of choice. Gas chromatography is used much more rarely, because most of the drugs possess limited volatility—in such cases derivatization (transformation into more volatile substance) may be necessary. Such procedure was used for example for determination of diazepam and carbamazepine (*N*-methyl-*N*-(trimethylsilyl) trifluoroacetamide was applied as a derivatizing agent) (Dȩbska et al. 2004). However, in the case of relatively non-polar pharmaceuticals, GC–MS without derivatization may be used (for instance in multi-residue analysis of pharmaceuticals, including carbamazepine, imipramine, dezipramine, doxepin, diazepam, and nordiazepam, in environmental samples (Togola and Budzinski 2008)). Besides separation, the choice of the proper detection method is very important. UV detection, which was formerly frequently used, possesses two main disadvantages. Firstly, it does not give any information about potential degradation product structure. Because of this limitation, it can be used only for quantitative analysis. Moreover, not every compound possesses chromophore regions that enable its determination with use of a UV detector. Taking this into account, nowadays, other detectors are preferred, especially the MS detector, which gives information about the exact mass of the compound (high-resolution mass spectrometry HR-MS) and even its fragments (tandem mass spectrometry or MS^n^). Of course, this kind of detector is also not free from disadvantages—it offers relatively low sensitivity in the case of poorly ionizing molecules and often allows identification of only probable structure of the analyzed compound—that is why sometimes NMR or IR detectors are preferred. But, what has to be admitted, they are also much less sensitive than MS. What is more, unless hyphenated LC–IR or LC–NMR techniques are applied, they demand pre-isolation of examined substances, which is often impossible because of very low concentration of degradation products. Another problem frequently encountered in the case of MS detectors is their susceptibility to the matrix effect, especially when the ESI ion source is used. This problem refers especially to environmental samples and could be solved by proper sample preparation, such as application of sample filtration (glass filers, syringe filters) and extraction (Ramirez et al. 2007), especially SPE (but LLE extraction is also used in some cases; Calisto and Esteves 2009). This technique enables not only reduction of matrix effects but also enrichment of studied samples, which is important because of very low concentrations of the pharmaceutical in the environment. Another group of methods gaining popularity are those based on immunoassay tests. Because of their specificity, they can be successfully used even in the case of very complex matrices. However, immunoassay tests such as radioimmunoassay, enzyme immunoassay, or chemiluminescence immunoassay, were applied to determination of psychotropic drugs in environmental samples (Silva et al. 2015); they are not popular in photodegradation research. Analytical methods used in photodegradation studies on psychotropic pharmaceuticals (based on data assembled in Tables 1, 2, and 3) are presented in Fig. 26.

Summing up, liquid chromatography linked with mass spectrometry detection is the most powerful analytical technique in photodegradation studies. The best performance can be achieved using ultra-high-performance chromatography (increased resolution and shortened time of analysis in comparison to regular HPLC), a diode array detector (measurement of absorption at various wavelengths is useful to detect molecules differing in absorption maxima; another advantage of an on-line DAD detector is the possibility of detection of poorly ionizing compounds that cannot be detected by an MS detector, and simultaneous quantitative and qualitative analysis when the MS detector works in dynamic range mode—which results in reduction of time and cost of analysis), and a high-resolution mass spectrometer (Q-TOF, Orbitrap), especially MS/MS enabling ion fragmentation. An instrument such this allows performing complex analysis of the photodegradation process: reaction kinetics as well as elucidation of degradation product structures. One of the most important future challenges is to make elucidation of phototransformation product structures and their toxicity assessment routine procedure. The importance of such research is supported by conclusions of many studies in which photodegradants turned out to be more toxic than parent compounds. However typical stability-indicating methods are still popular these days, use of high-resolution multi-stage mass spectrometry is becoming an apparent trend.

## Conclusion

Psychotropic drugs are nowadays one of the most frequently used group of pharmaceuticals, and, according to predictions, their consumption will be increasing, especially in high-income countries. Moreover, treatment of mental diseases usually demands chronic, often combined, therapy. These facts make safety of psychotropic drugs substantial. Taking into account that ultraviolet and visible radiation are very common stressing factors—on every step of manufacturing and shelf-life, or even after ingestion of drug—assessment of photostability and photodegradation process cannot be underestimated. Unfortunately, guidelines presented by the ICH Q1B document applied in many countries, are sometimes unclear, especially in the case of suggested light source options, which are in fact not equivalent. This may be considered as one of the causes of various, sometimes completely differing, conditions applied in photostability/photodegradation studies on psychotropic drugs and, as an effect, incoherent as well as unreliable findings.

High consumption of psychiatric pharmaceuticals leads also to their accumulation in the environment. Ecotoxicity of some of them has been confirmed; hence, notable amounts found in wastewater effluents, municipal wastes, groundwater, or even lake and seawater pose a threat to wildlife. Still commonly used traditional wastewater treatment methods are often ineffective; hence, necessity of researches on advanced procedures is obvious. The new methods have to be not only effective in removal of pharmaceuticals (and their metabolites) but also apply relatively low toxic chemicals and, what is not less important, they should be acceptable from the economic point of view. Advanced oxidation processes using various titanium dioxide photocatalysts seem to fulfill these requirements. Similarly to studies on photodegradation, researches on new wastewater treatment methods sometimes leave a lot to be desired—for example application of UV-C radiation sources or use of methanolic solutions, which is irrelevant from an environmental point of view. What is more, frequently used quantitative chromatographic assay or a measure of total organic carbon is insufficient because of the possible creation of a compound much more toxic than the parent molecule, and even a genotoxic or carcinogenic species. Even traces of such substances are potentially harmful, especially in the case of long-time exposure. That is why identification of photodegradation product structures is very crucial. The problem of formation of toxic photoproducts may be solved by introduction of radiation—molecule interaction investigation during the design stage of the new drugs. A very important and often neglected question is exhaustive photostability testing of pharmaceuticals present on the market. Such studies should absolutely include structural elucidation and, preferably, toxicity assessment of the photoproducts.

Many analytical methods were used for the photodegradation studies of psychotropic drugs; however, chromatographic, spectroscopic and spectrometric methods were the most often applied. In should be noticed that the combined techniques such as LC and GC combined with multi-stage mass spectrometry, and LC-NMR turned out to be the most powerful analytical tools in this case. These methods allow performing a complete analysis of the photodegradation process including the determination of kinetics reaction as well as elucidation of chemical structures of degradation products. Spectra libraries (MS/MS) concerning pharmaceuticals and related photoproducts would facilitate determination of potentially toxic products of phototransformation.

2-PrOH, propan-2-ol; ANAD, anaerobic degradation test (ISO 11734:1995); AOP, advanced oxidation process; AOS, active oxygen species; ATC, anatomical therapeutic chemical classification system; CBT, closed bottle test (OECD 301 D); DOM, dissolved organic matter; EAS, electron absorption spectrometry; EPR, electron paramagnetic resonance; EtOH, ethanol; GC, gas chromatography; GLYC, glycogen content; GSTs, glutathione transferases; HPLC, high-performance liquid chromatography; HPTLC, high-performance thin-layer chromatography; HW, humic water; IC, ion chromatography; IR, infrared spectroscopy; IT ion trap; LBT, luminescent bacteria test; LC, liquid chromatography; LDH, lactate dehydrogenase; LPO, lipid peroxidation; MAO, monoamine oxidase; MEKC, micellar electrokinetic chromatography; MetOH, methanol; MRT, manometric respiratory test (OECD 301F); MS, mass spectrometry; NMR, nuclear magnetic resonance spectroscopy; NOM, natural organic matter; PDA, photodiode array detector; PG, propylene glycol; PrOH, propan-1-ol; PROT, protein content; SAXS, small-angle X-ray scattering; SFW, synthetic field water; SHW, synthetic humic water; SOD, superoxide dismutase; SPE, solid-phase extraction; Specroph., spectrophotometry; SS, solid state; SSRI, selective serotonin reuptake inhibitors; TFA, trilfluoroacetic acid; THF, tetrahydrofuran; TLC, thin-layer chromatography; TOC, total organic carbon; UHPLC/UPLC, ultra-high-performance liquid chromatography; WW, wastewater; YES, yeast estrogen screen

## Footnotes

1 WHO defines the DDD as *the assumed average maintenance dose per day for a drug used on its main indication in adults.* Values of DDD vary between the psychotropic drug substances from, for instance, 0.7 mg for fluspirilene to 1200 mg for sultopride (typical and atypical antipsychotic drug respectively), thus representing their consumption in mass units could be difficult. However, if the DDD of psychotropic drugs arithmetic mean amounts to 146.20 mg, their annual consumption equals 61.40 tonnes.

2 Although carbamazepine is not classified as an antipsychotic, antidepressant, or anxiolytic drug, it was discussed in this review because of its use in the treatment of schizophrenia (in combination with antipsychotics) (Alrashood 2016) and common occurrence in the environment. The drug was attached to benzodiazepine-class pharmaceuticals.
